# Supplementary figures and images for: HAPLN2 forms aggregates and promotes microglial inflammation during brain aging in mice
Source: PLoS Biol. 2025 Aug 14;23(8):e3003006. doi: 10.1371/journal.pbio.3003006 (PMC12407547; doi:10.1371/journal.pbio.3003006)

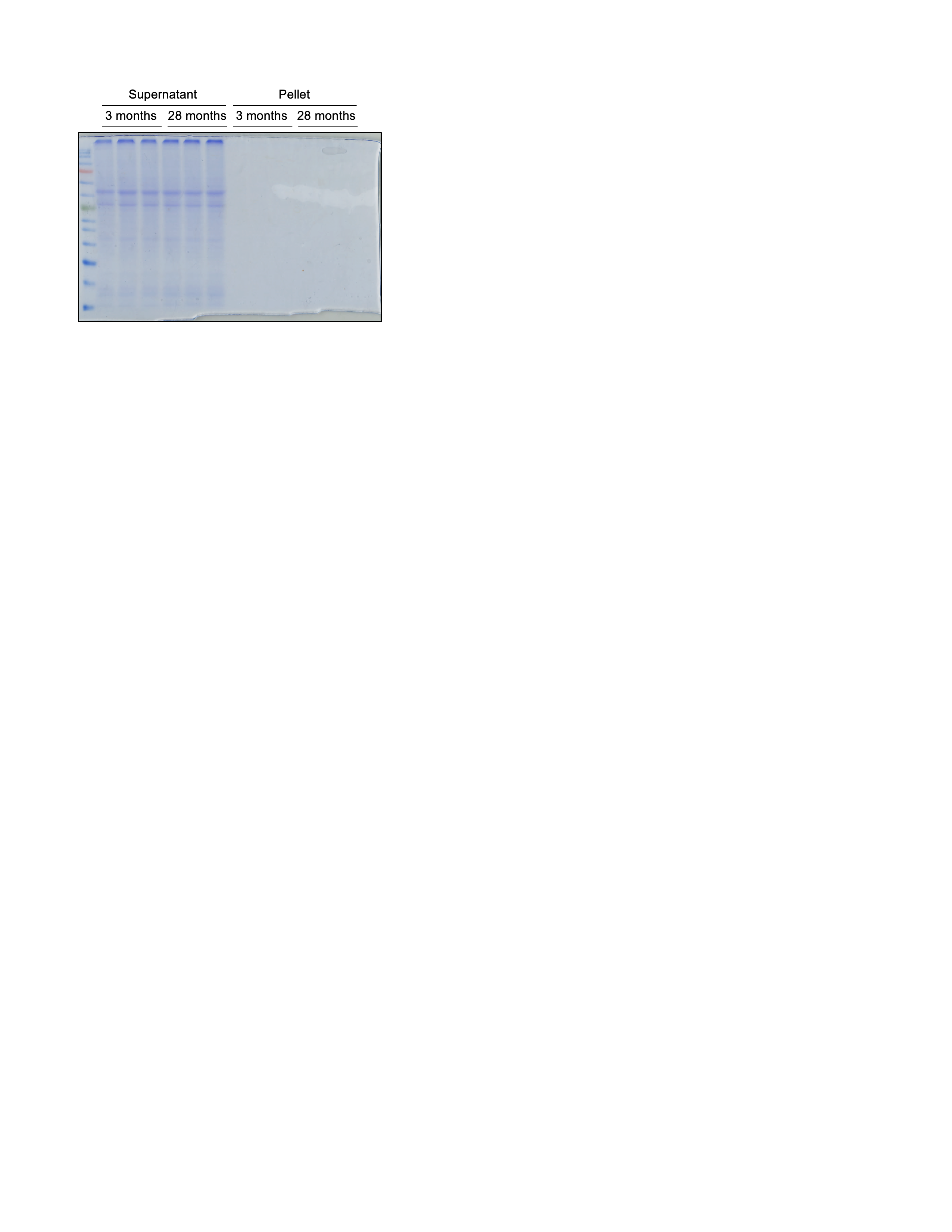

Supplement: S1 Fig — Coomassie brilliant blue staining of young (3-month-old) and aged (28-month-old) mouse brains. Whole brains were lysed with PTS buffer and centrifuged at 15,000 g. n = 3. (TIFF) [file pbio.3003006.s001.tiff]

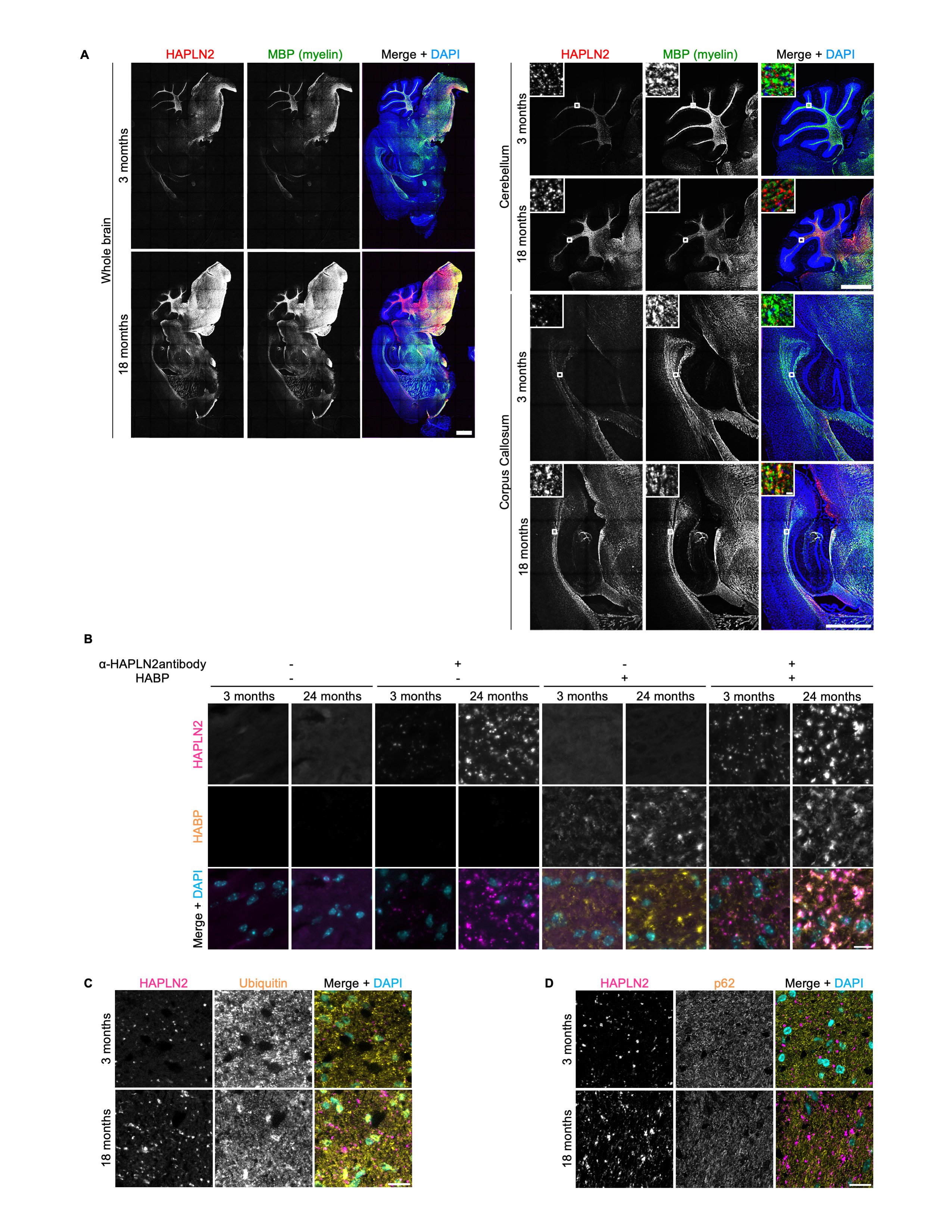

Supplement: S2 Fig — (A) Immunohistochemistry images of the mouse whole brain, cerebellum, and corpus callosum stained with anti-HAPLN2 antibody and anti-myelin basic protein (MBP) antibody. n = 3. (B) Magnified immunohistochemistry images of the mouse cerebellar white matter stained with anti-HAPLN2 antibody or biotinylated hyaluronic acid-binding protein (HABP), which probes hyaluronic acid. Specific staining of HAPLN2 and HABP was observed without crossover. n = 3. (C, D) Fluorescence immunohistochemistry images of the mouse cerebellar white matter showing staining for ubiquitin (C) and p62 (D). n = 3. Scale bars: 1 mm (A), 50 µm (B, C, D), and 10 µm (A (inlet)). (TIFF) [file pbio.3003006.s002.tiff]

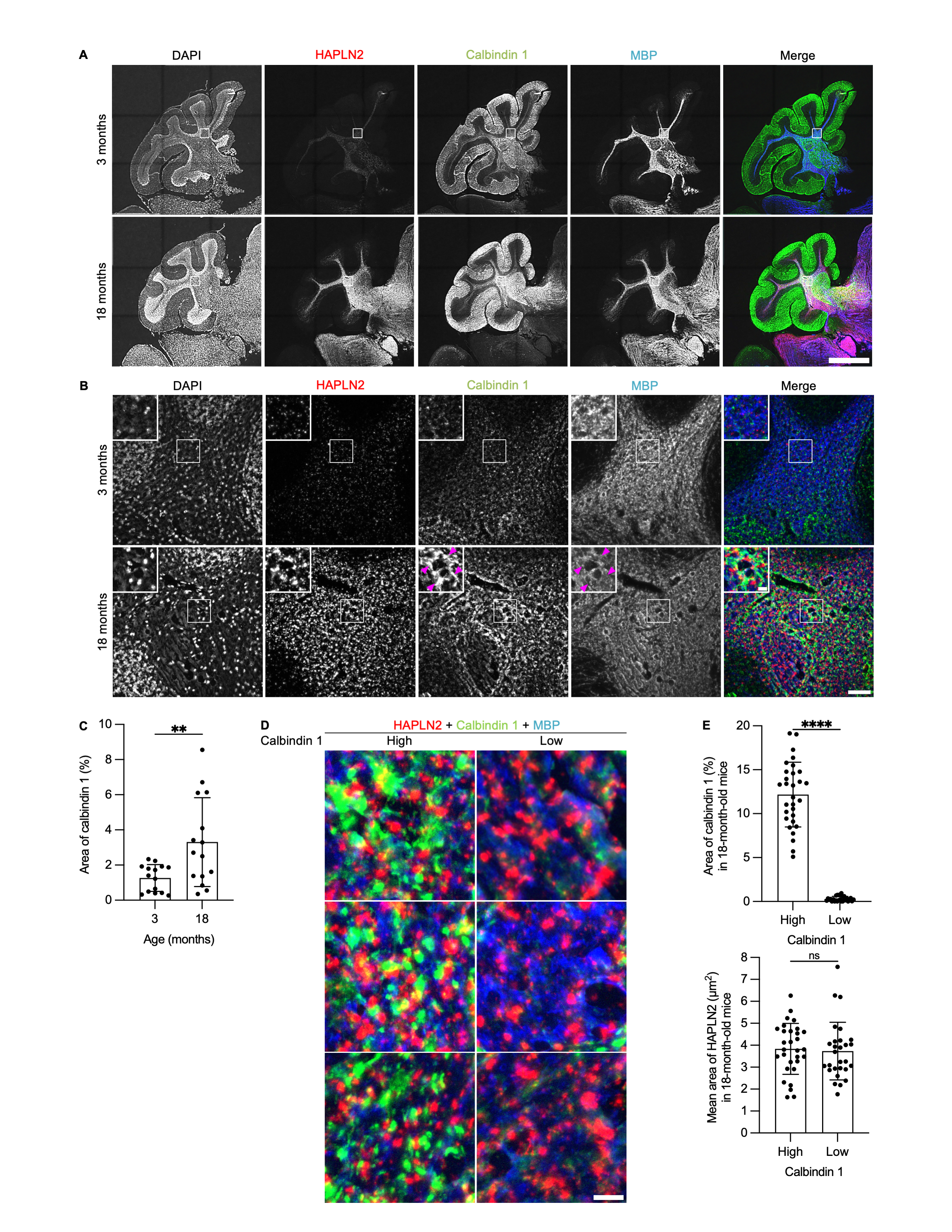

Supplement: S3 Fig — (A) Immunohistochemistry images of the mouse cerebellum stained with anti-HAPLN2 antibody, anti-calbindin 1 antibody, and anti-MBP antibody. n = 3. (B) Magnified fluorescence immunohistochemistry images for HAPLN2, calbindin 1, and MBP corresponding to the boxed areas in (A). Regions indicated by magenta arrows showed MBP staining voids accompanied by increased calbindin 1 staining. (C) Quantitation of the mean area of calbindin 1 in (B) comparing young (3-month-old) and aged (18-month-old) mice. (D) Magnified fluorescent immunohistochemistry images of calbindin 1–high and –low regions in the cerebellar white matter of 18-month-old mice. High and low regions were defined as those with more than 5% and less than 1% calbindin 1–positive area, respectively. (E) Quantitation of the mean area of calbindin 1 (left) and HAPLN2-positive puncta (right) in calbindin 1–high region and –low region in 18-month-old mice, as shown in (D). Scale bars: 1 mm (A), 50 µm (B), and 10 µm (B (inlet), D). Error bars represent mean ± S.D. P-values were calculated using two-tailed Student t test. **p < 0.01, ****p < 0.0001, ns = not significant. The underlying data for (C) and (E) can be found in S1 Data. (TIFF) [file pbio.3003006.s003.tiff]

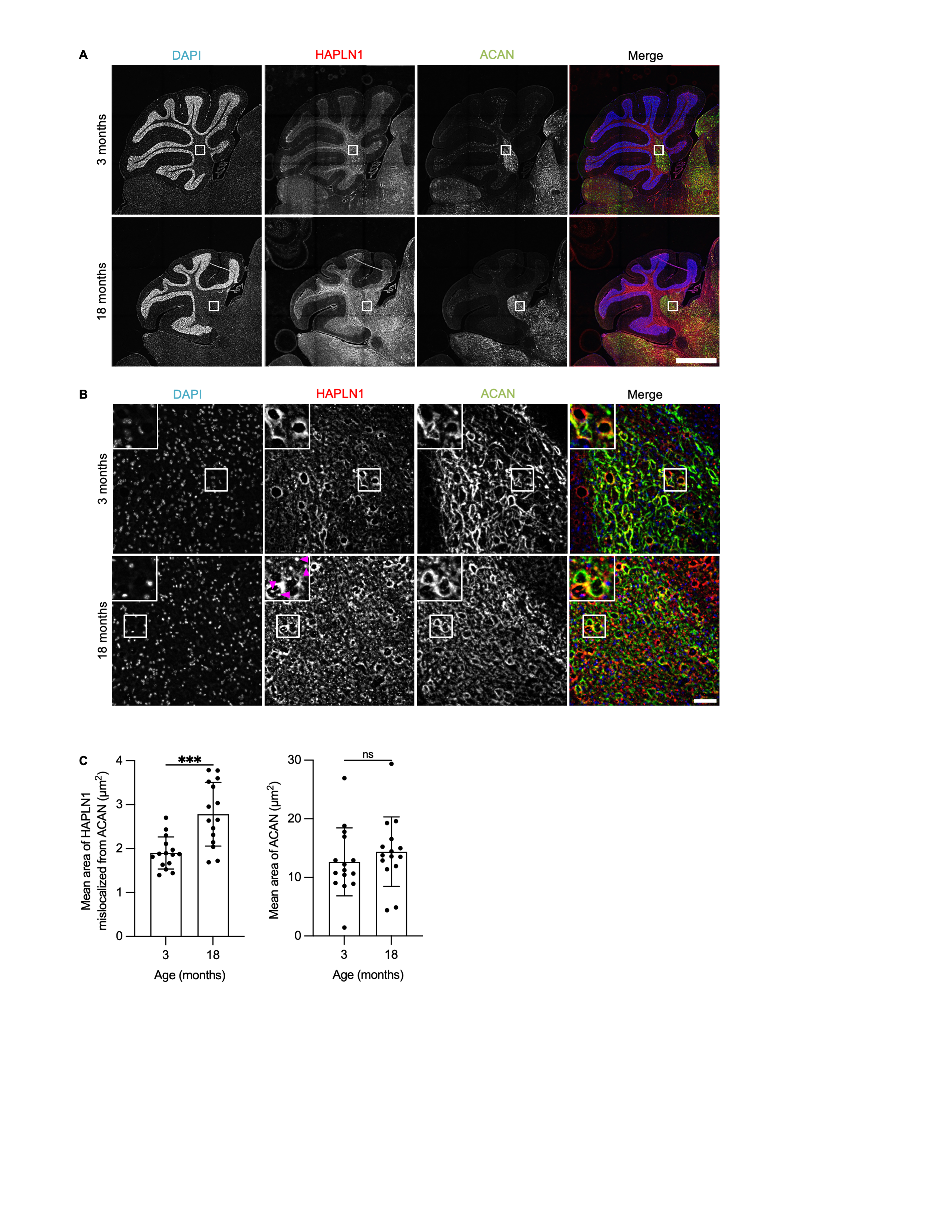

Supplement: S4 Fig — (A) Immunohistochemistry images of the mouse cerebellum stained with anti-HAPLN1 antibody and anti-ACAN antibody. n = 3. (B) Magnified fluorescence immunohistochemistry images for HAPLN1 and ACAN corresponding to the boxed areas in (A). The magenta arrows indicate HAPLN1 that are mislocalized relative to ACAN staining. (C) Quantitation of the mean area of HAPLN1 (left) and ACAN-positive area (right) in (B). The underlying data can be found in S1 Data. Scale bars: 1 mm (A), 50 µm (B). Error bars represent mean ± S.D. P-values were calculated using two-tailed Student t test. ***p < 0.001, ns = not significant. (TIFF) [file pbio.3003006.s004.tiff]

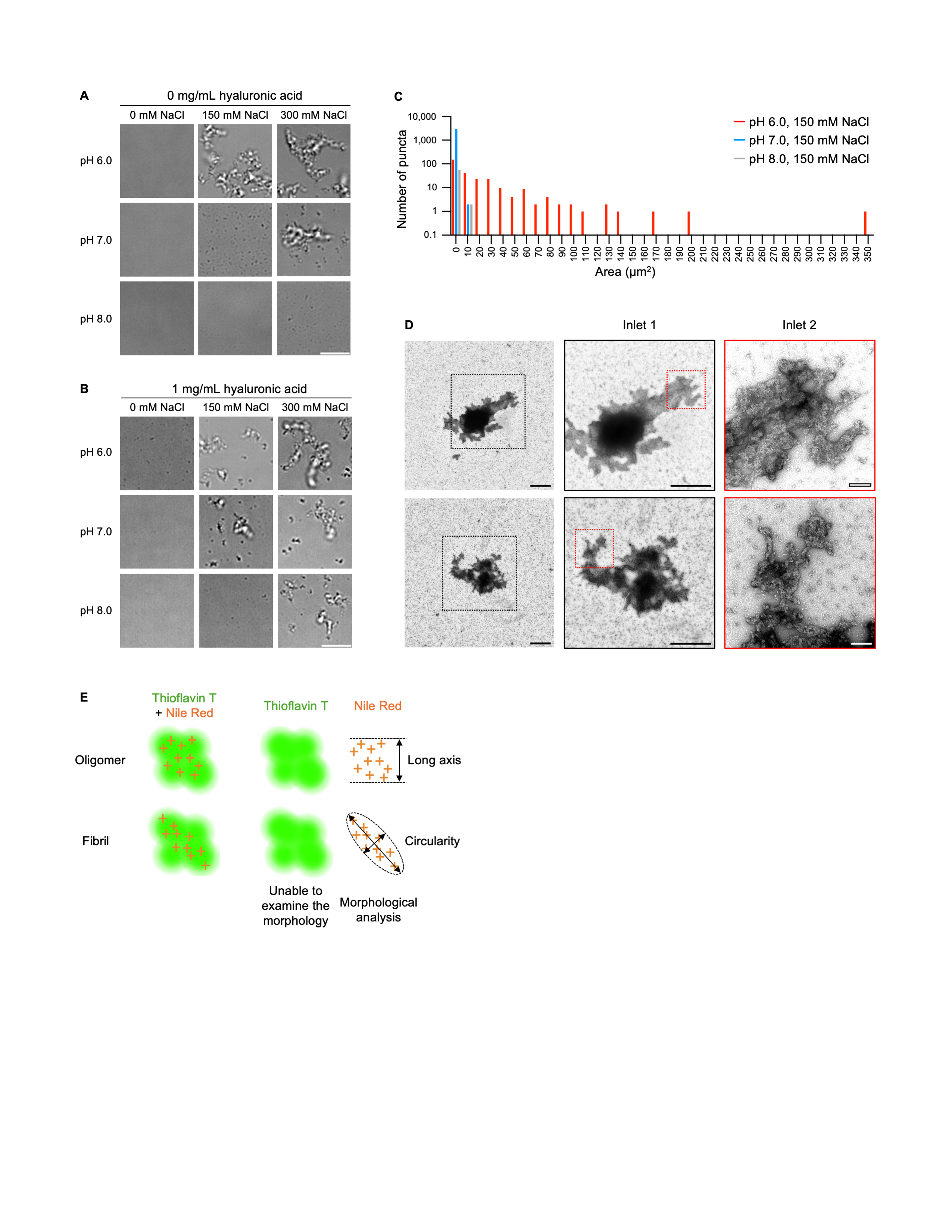

Supplement: S5 Fig — (A, B) Brightfield microscopic images showing aggregates of recombinant HAPLN2 incubated under various buffer conditions in the absence (A) or presence (B) of hyaluronic acid. Data were collected from at least five fields of view. (C) Histogram depicting the area of individual aggregates of recombinant HAPLN2 after 24 hours of incubation in the indicated buffer. Aggregate areas were measured using ImageJ (version 1.53t). The underlying data can be found in S1 Data. (D) Transmission electron microscopy images of recombinant HAPLN2 aggregates formed under 150 mM NaCl (pH 6.0). Data were collected from at least five fields of view. (E) S chematic representation of cross-β-sheet-positive oligomers and fibrils stained with thioflavin T and Nile Red. Created with BioRender.com. Scale bars: 10 µm (A, B), 1 µm (D, black), and 100 nm (D, white). (TIFF) [file pbio.3003006.s005.tiff]

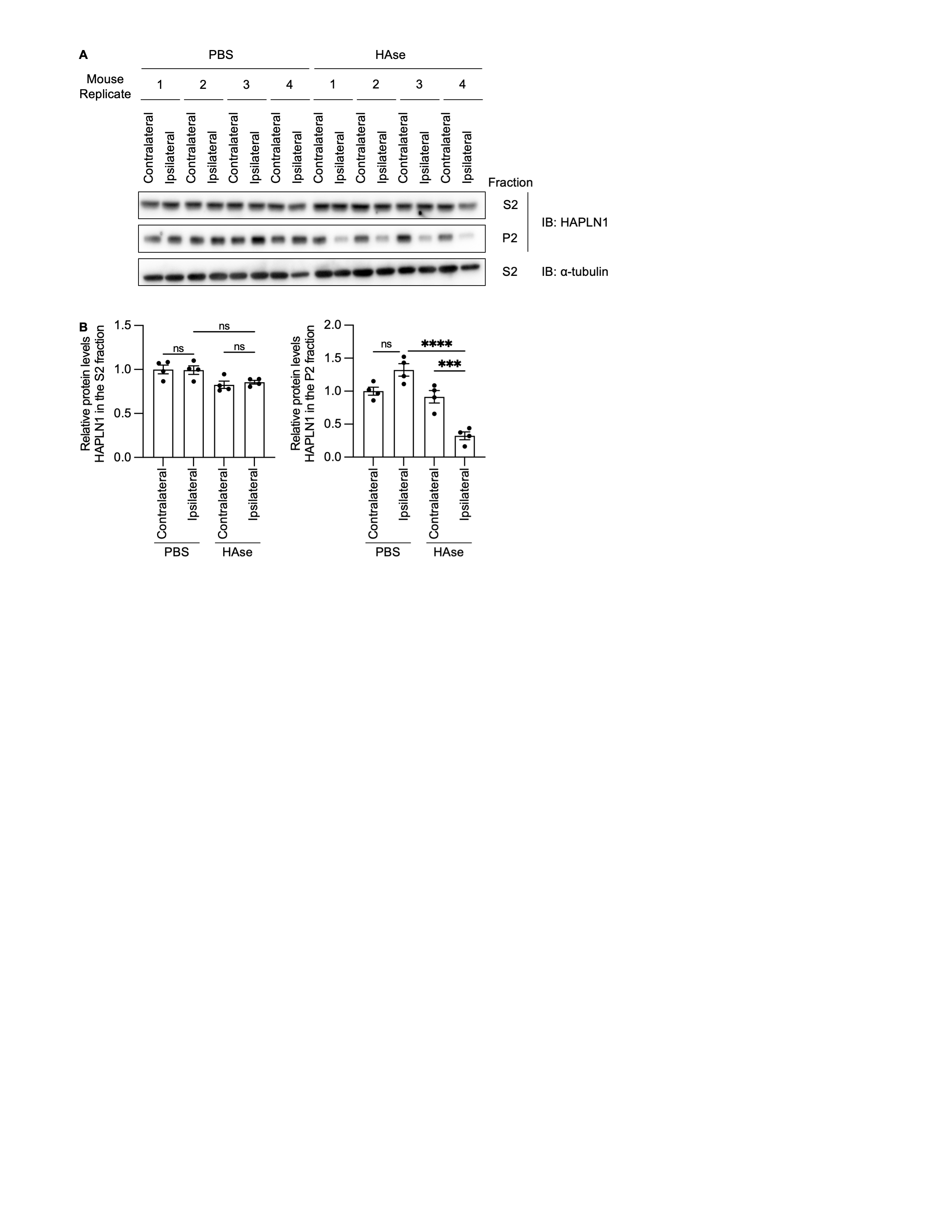

Supplement: S6 Fig — (A) Immunoblot analysis of the cerebellum corresponding to the samples shown in Fig 6D. n = 4. (B) Densitometric quantification of (A). HAPLN1 protein levels in the S2 fraction were normalized to α-tubulin in the S2 fractions. HAPLN1 protein levels in the P2 fraction were normalized to α-tubulin in the S1 fractions. n = 4. The underlying data can be found in S1 Data. Error bars represent mean ± S.E.M. P-values by one-way ANOVA followed by Tukey’s post hoc test. ***p < 0.001, ****p < 0.0001, ns = not significant. (TIFF) [file pbio.3003006.s006.tiff]

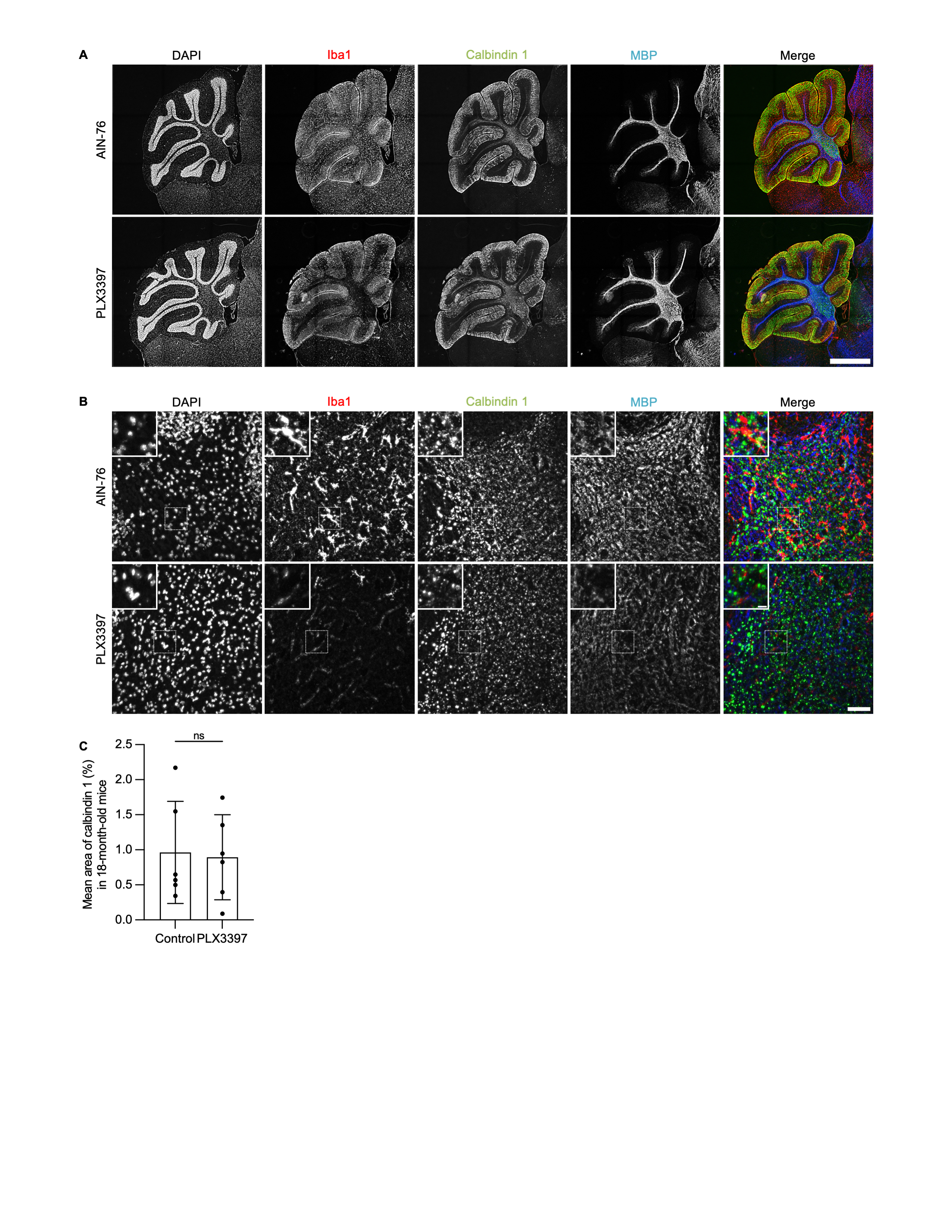

Supplement: S7 Fig — The mean area of calbindin 1 did not show a significant difference after PLX3397 treatment. (A) Immunohistochemistry images of the mouse cerebellum stained with anti-Iba1 antibody, anti-calbindin 1 antibody, and anti-MBP antibody. n = 3. (B) Magnified fluorescence immunohistochemistry images for Iba1, calbindin 1, and MBP corresponding to the boxed areas in (A). (C) Quantitation of the mean area of calbindin 1 (B). The underlying data can be found in S1 Data. Scale bars: 1 mm (A), 50 µm (B), and 10 µm (B, inlet). Error bars represent mean ± S.D. P-values were calculated using two-tailed Student t test. ns = not significant. (TIFF) [file pbio.3003006.s007.tiff]

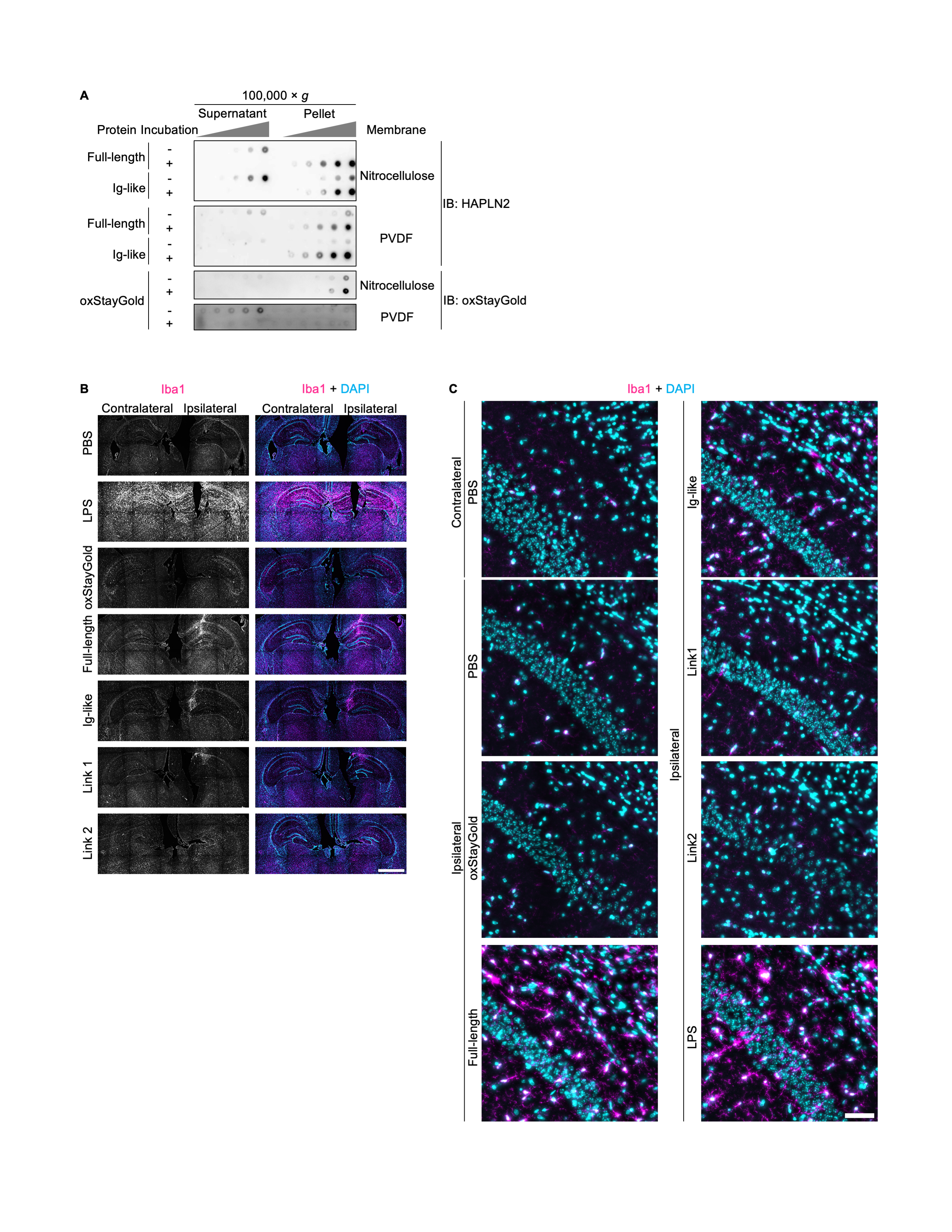

Supplement: S8 Fig — (A) Filter trap assay and immunoblot analysis of recombinant full-length HAPLN2, HAPLN2 Ig-like domain, and oxStayGold proteins. These recombinant proteins (2 µM each) were incubated overnight at 37°C, followed by centrifugation at 100,000 g for one hour at 4°C. (B) Immunohistochemistry images of the coronal brain section from Fig 8E, stained with anti-Iba1 antibody to probe activated microglia. (C) Magnified fluorescence immunohistochemistry images for Iba1, corresponding to Fig 8D. Scale bars: 500 µm (B) and 50 µm (C). (TIFF) [file pbio.3003006.s008.tiff]
