# Supplementary material for: HAPLN2 forms aggregates and promotes microglial inflammation during brain aging in mice
Source: PLoS Biol. 2025 Aug 14;23(8):e3003006. doi: 10.1371/journal.pbio.3003006 (PMC12407547; doi:10.1371/journal.pbio.3003006)
Supplement: S1 File — Raw images for all IB. (PDF) [file pbio.3003006.s018.pdf]

Figure 2A

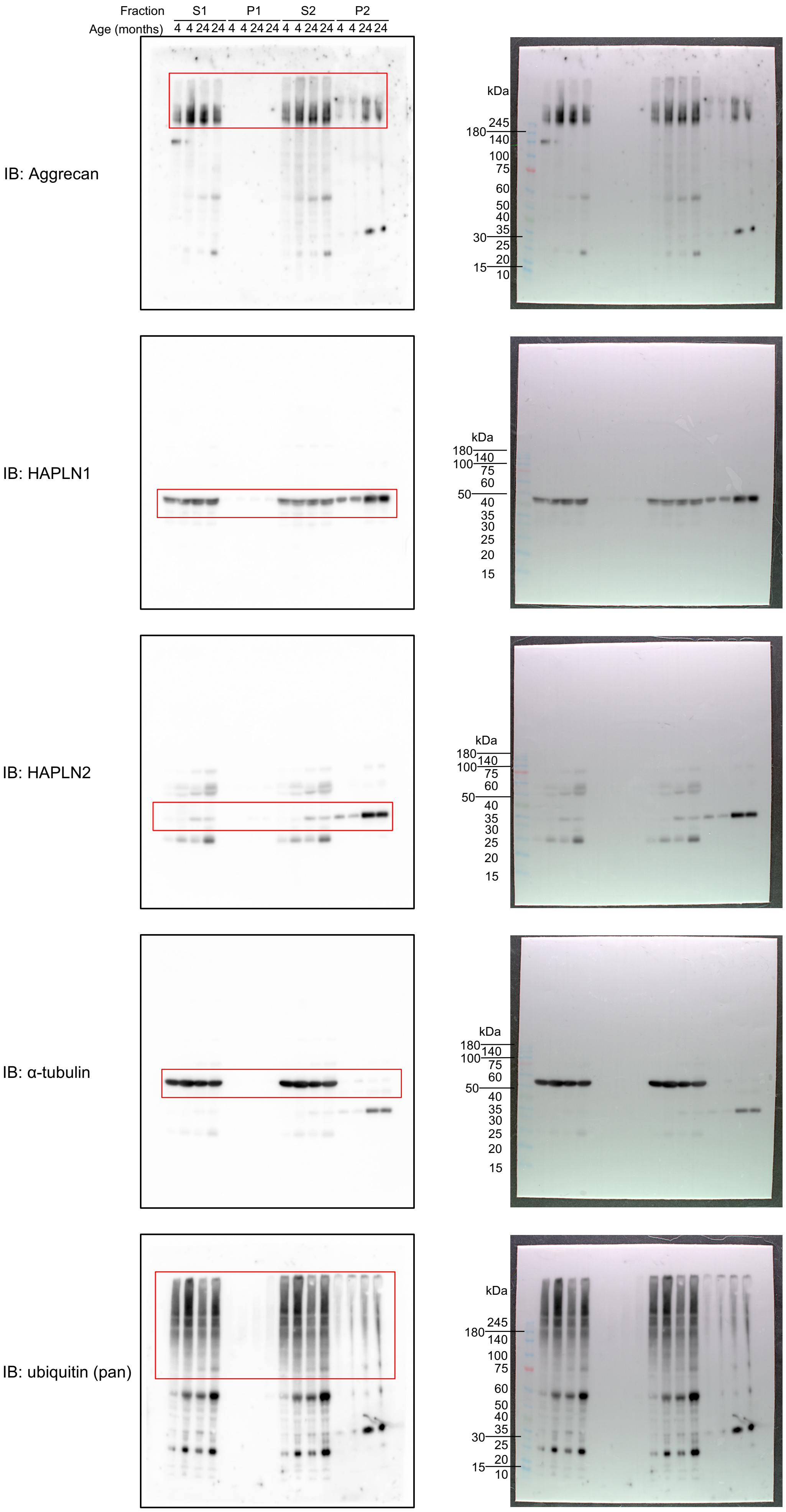

Fraction S1 P1 S2 P2
Age (months) 4 4 24 24 4 4 24 24 4 4 24 24 4 4 24 24
IB: Aggrecan
IB: HAPLN1
IB: HAPLN2
IB: α-tubulin
IB: ubiquitin (pan)
kDa
245
180
140
100
75
60
50
40
35
30
25
20
15
10

Figure 2C

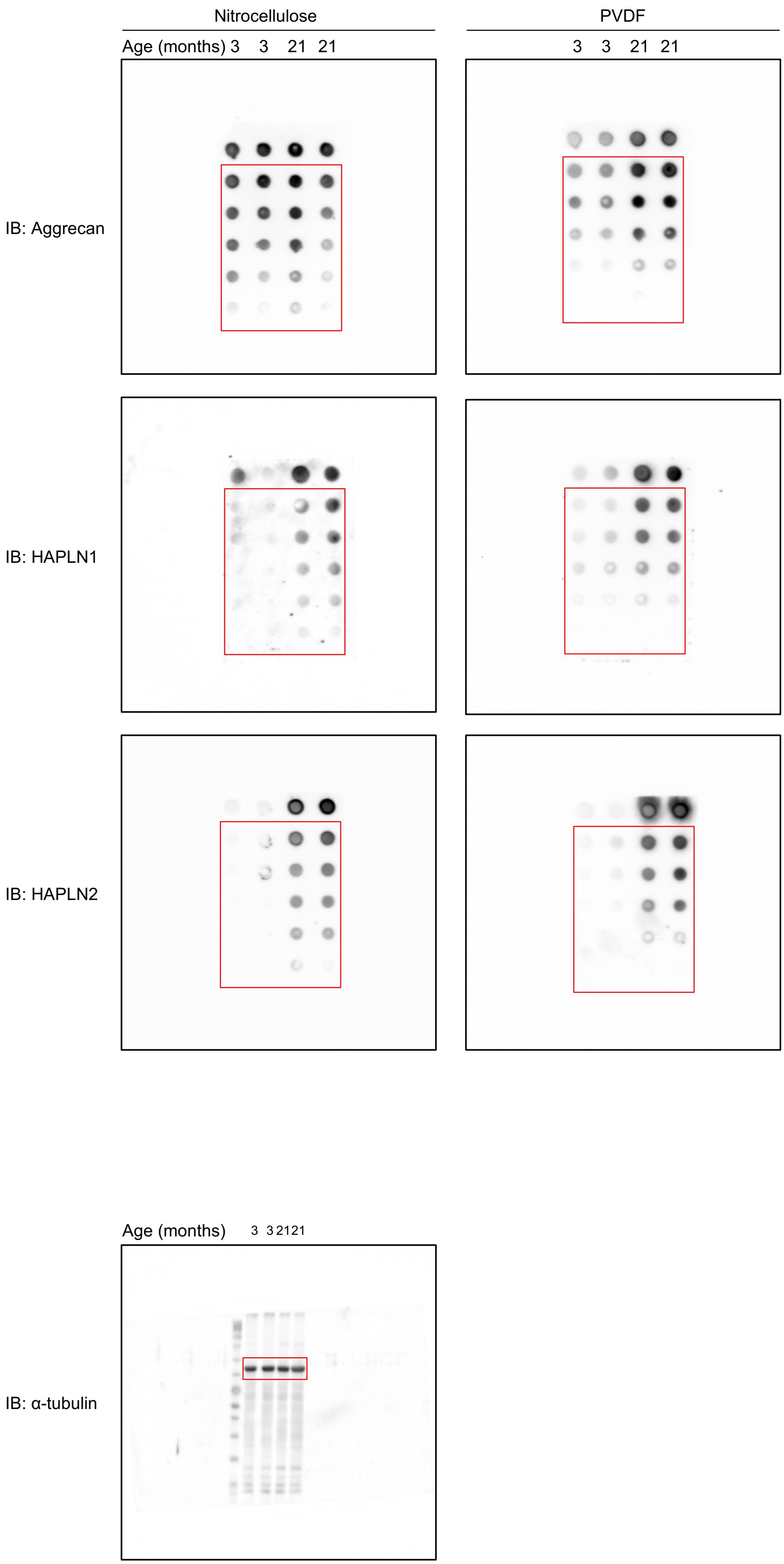

Nitrocellulose
PVDF
Age (months) 3 3 21 21
3 3 21 21
IB: Aggrecan
IB: HAPLN1
IB: HAPLN2
Age (months) 3 3 21 21
IB: α-tubulin

Figure 2E

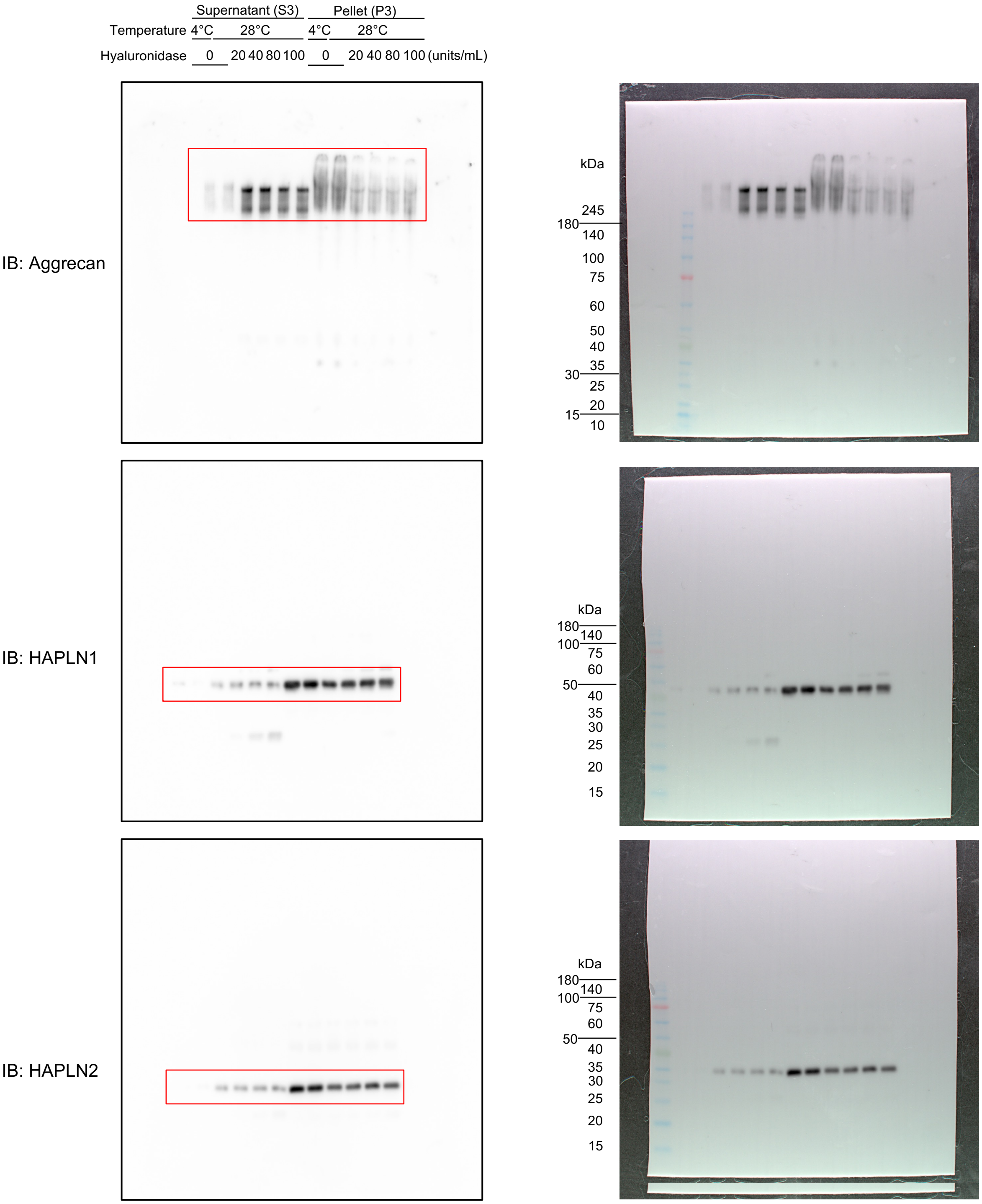

Supernatant (S3)
Pellet (P3)
Temperature 4°C 28°C 4°C 28°C
Hyaluronidase 0 20 40 80 100 0 20 40 80 100 (units/mL)
IB: Aggrecan
IB: HAPLN1
IB: HAPLN2
kDa
245
180
140
100
75
60
50
40
35
30
25
20
15
10

Figure 2F

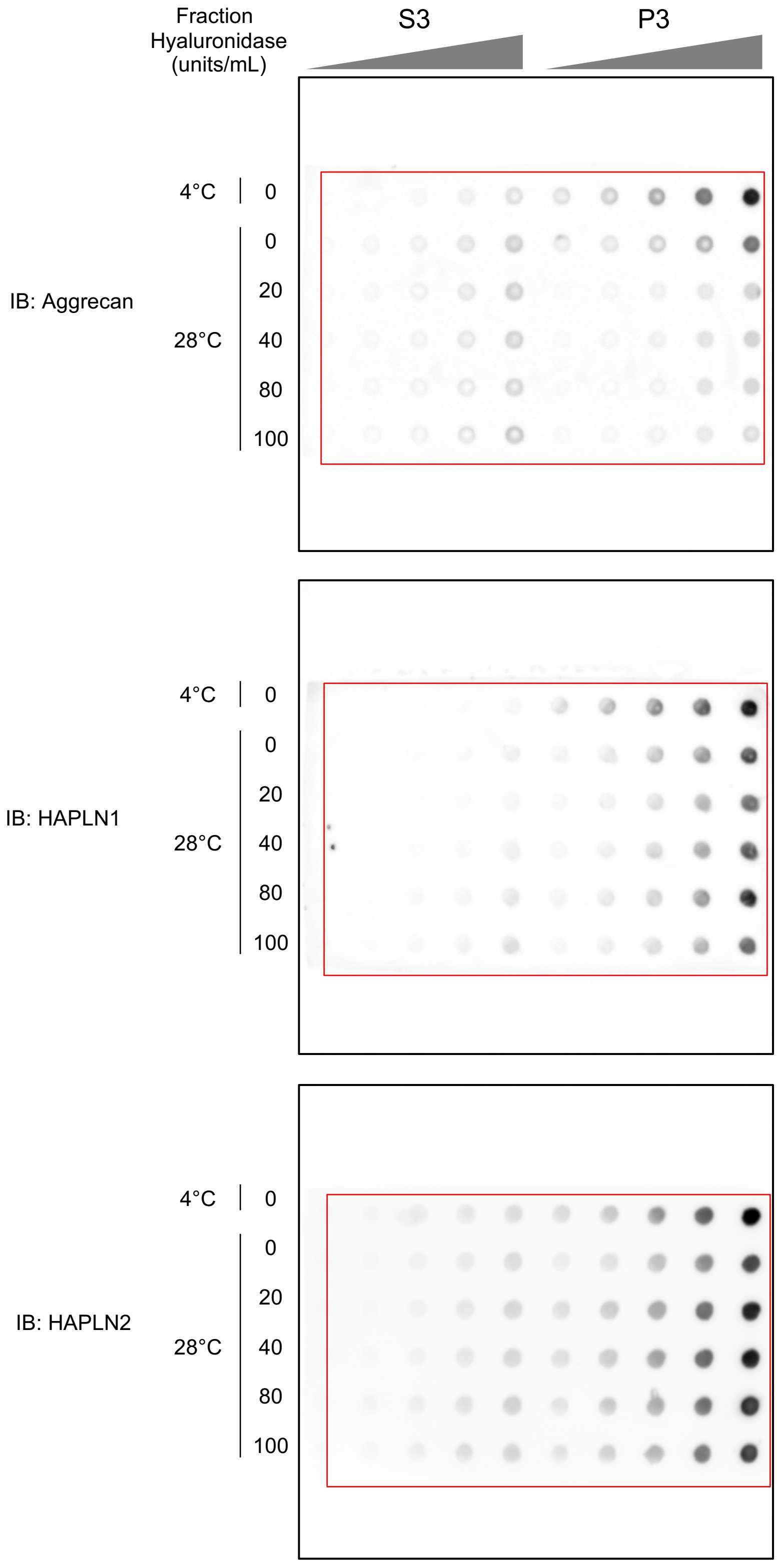

Fraction
Hyaluronidase
(units/mL)
S3
P3
4°C
0
0
20
IB: Aggrecan
28°C
40
80
100
4°C
0
0
20
IB: HAPLN1
28°C
40
80
100
4°C
0
0
20
IB: HAPLN2
28°C
40
80
100

Figure 3A

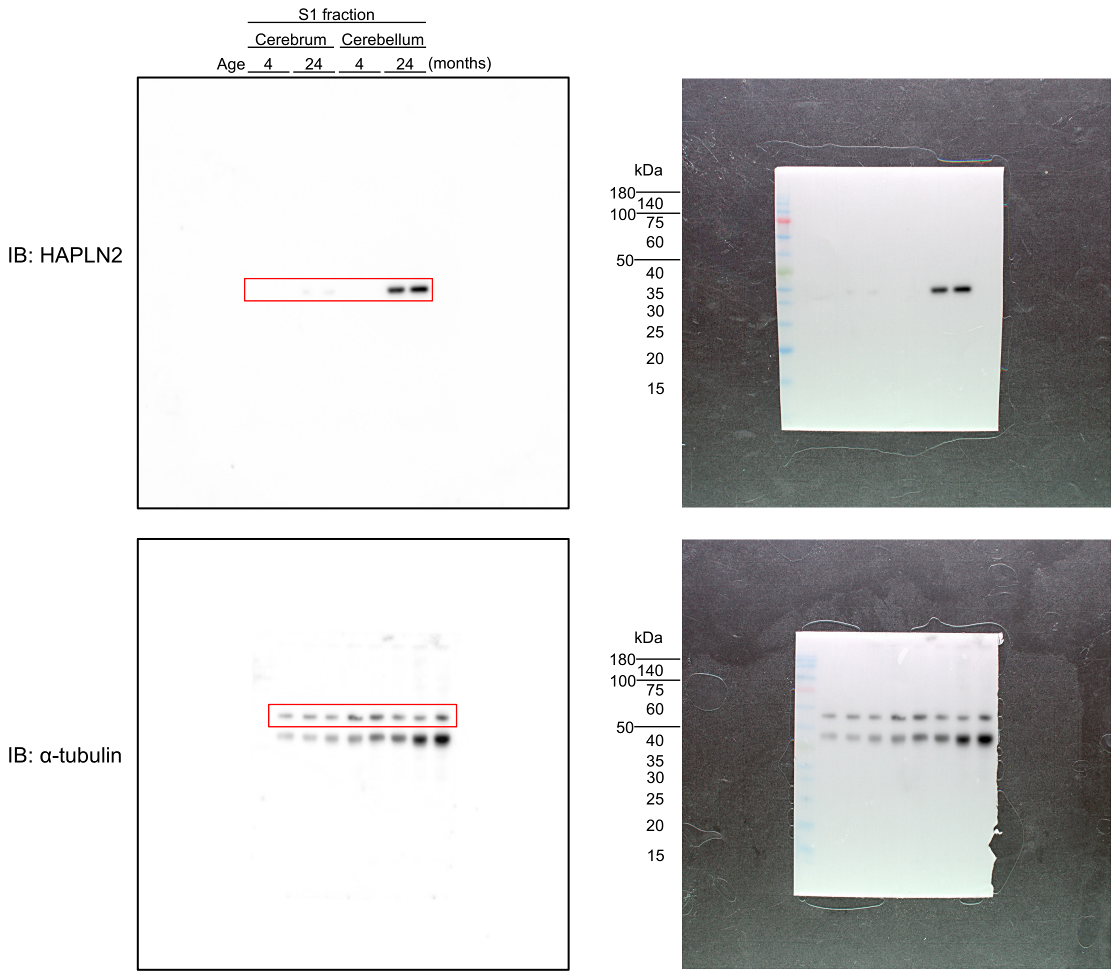

S1 fraction
Cerebrum
Cerebellum
Age 4 24 4 24 (months)
IB: HAPLN2
IB: α-tubulin
kDa
180
140
100
75
60
50
40
35
30
25
20
15
kDa
180
140
100
75
60
50
40
35
30
25
20
15

Figure 4A

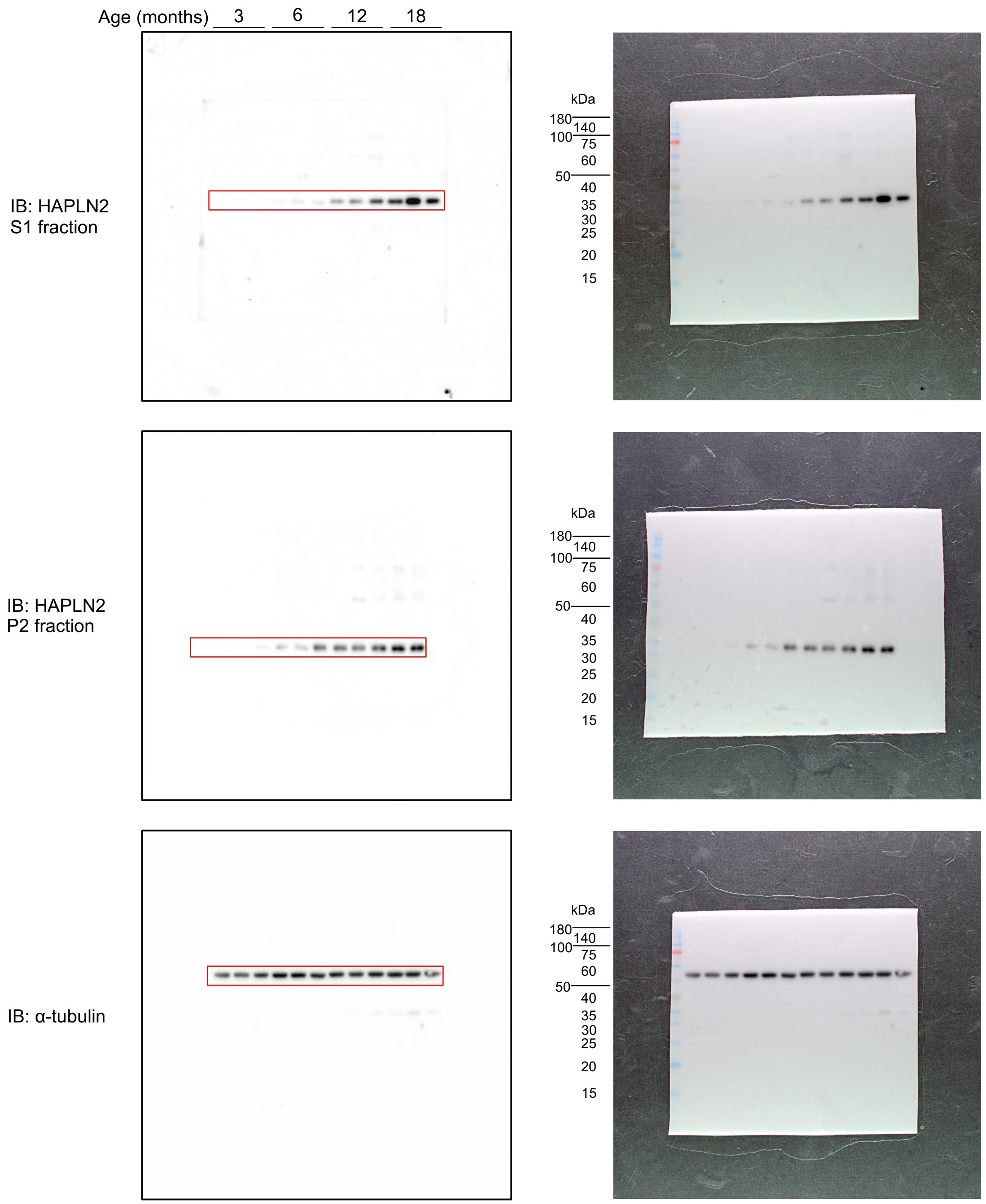
Age (months)
3
6
12
18
IB: HAPLN2
S1 fraction
IB: HAPLN2
P2 fraction
IB: α-tubulin
kDa
180
140
100
75
60
50
40
35
30
25
20
15

Figure 6D

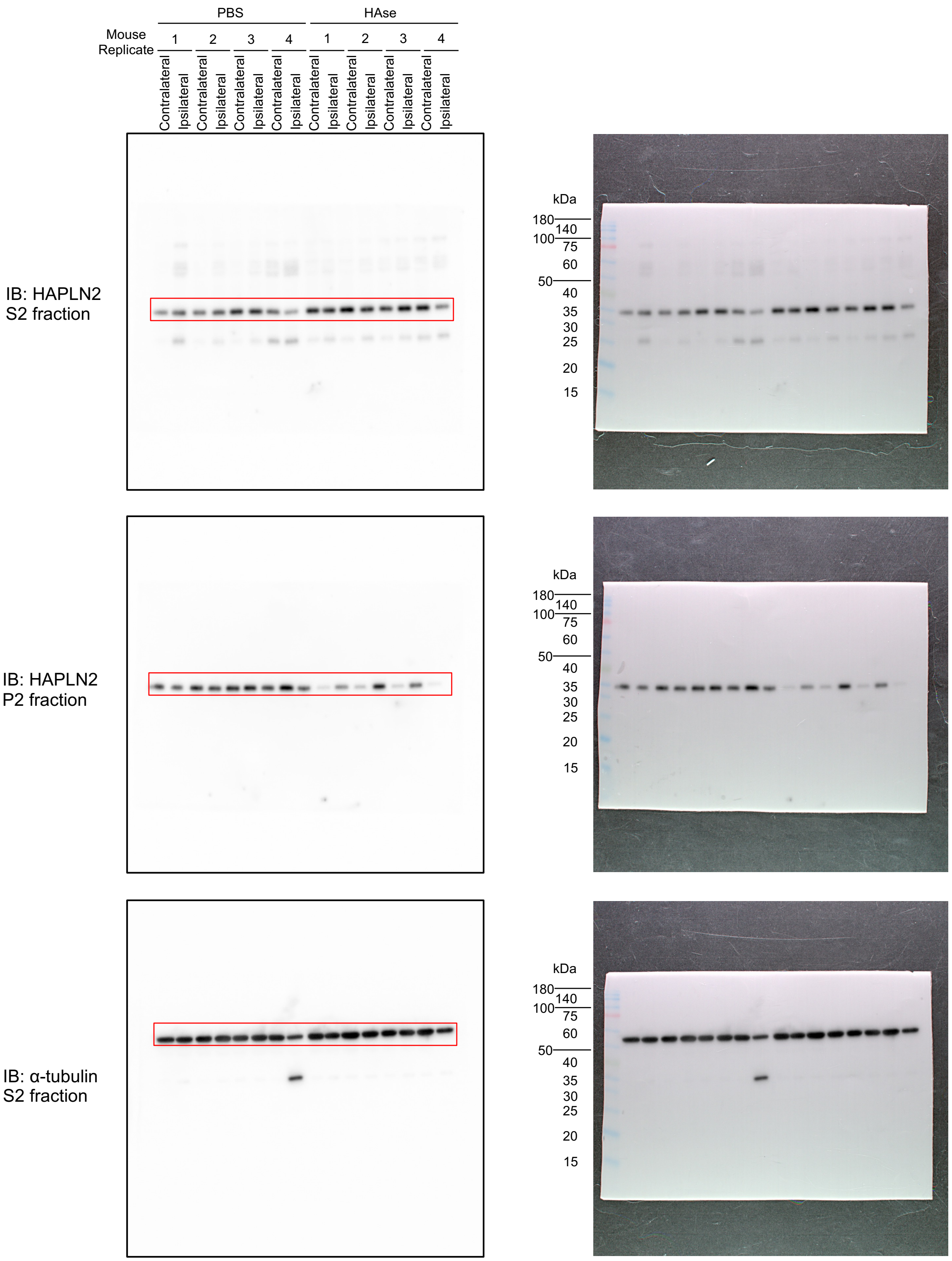

PBS
HAse
Mouse Replicate
1
2
3
4
1
2
3
4
Contralateral
Ipsilateral
IB: HAPLN2 S2 fraction
IB: HAPLN2 P2 fraction
IB: α-tubulin S2 fraction
kDa
180
140
100
75
60
50
40
35
30
25
20
15

Figure 6F

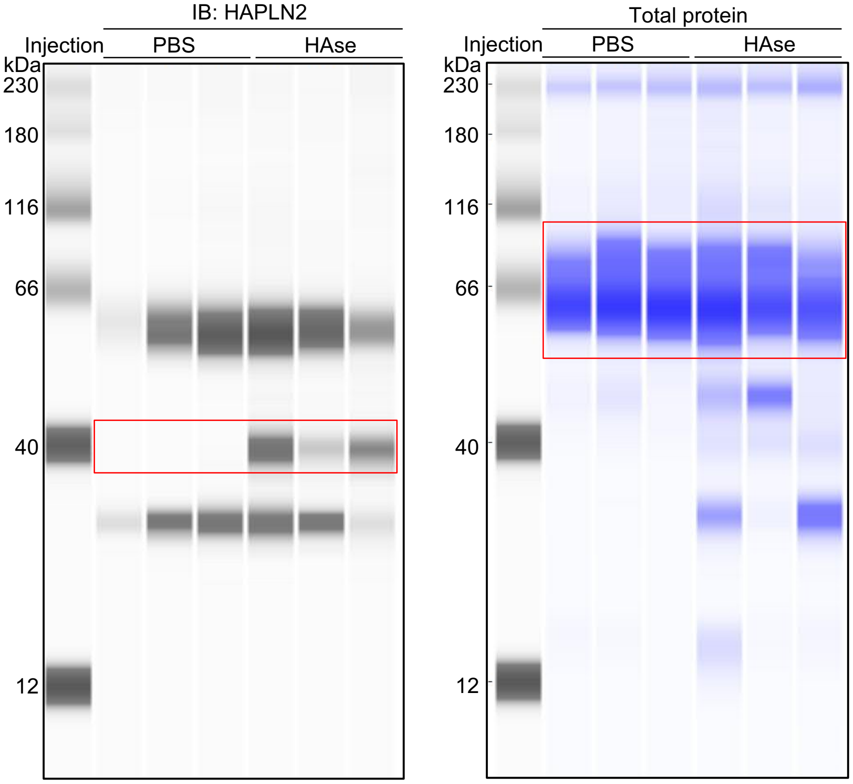
IB: HAPLN2
Injection
PBS
HAse
kDa
230
180
116
66
40
12
Total protein
Injection
PBS
HAse
kDa
230
180
116
66
40
12

Figure 7C

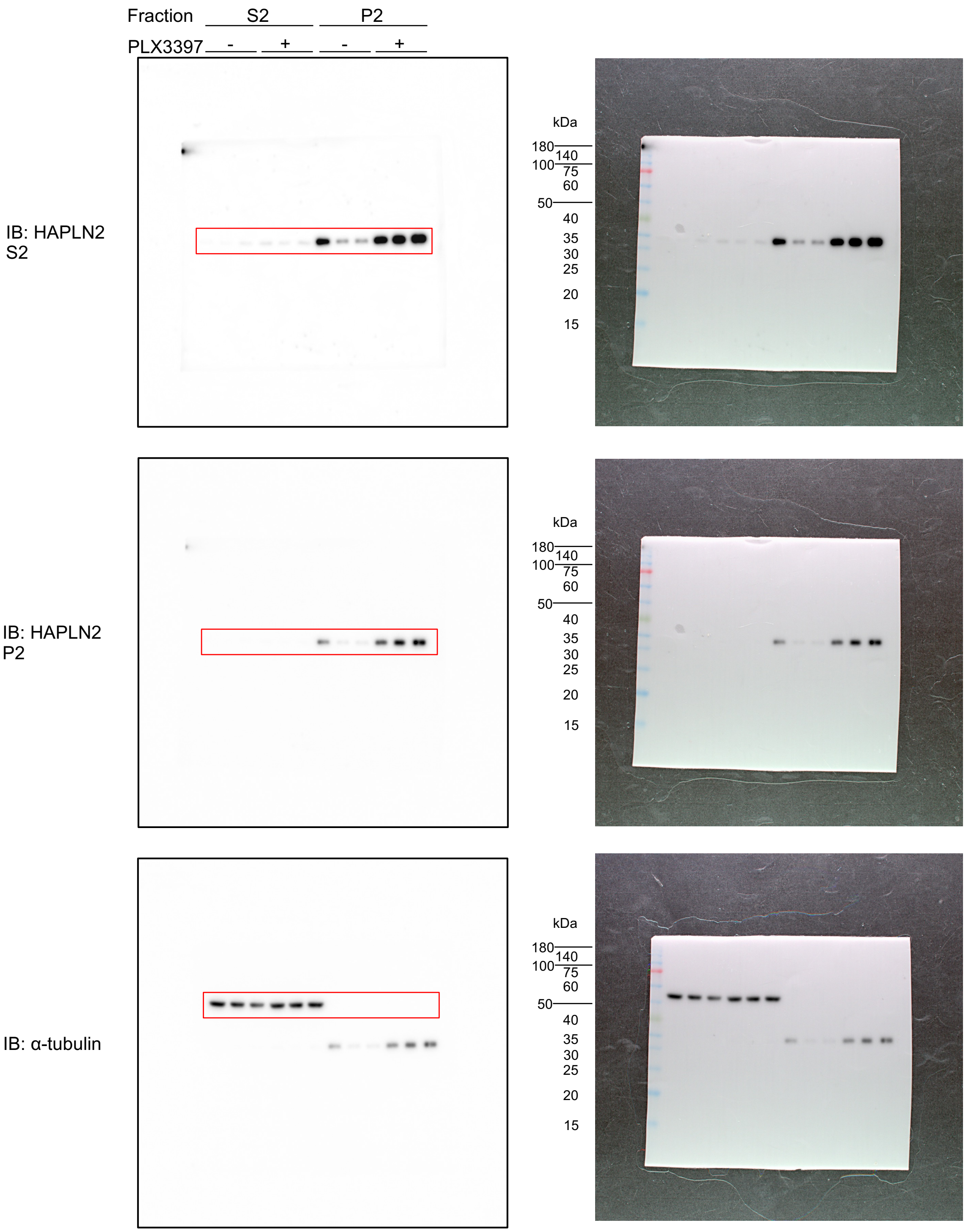
Fraction
S2
P2
PLX3397
-
+
-
+
IB: HAPLN2
S2
IB: HAPLN2
P2
IB: α-tubulin
kDa
180
140
100
75
60
50
40
35
30
25
20
15
kDa
180
140
100
75
60
50
40
35
30
25
20
15
kDa
180
140
100
75
60
50
40
35
30
25
20
15

Figure S6A

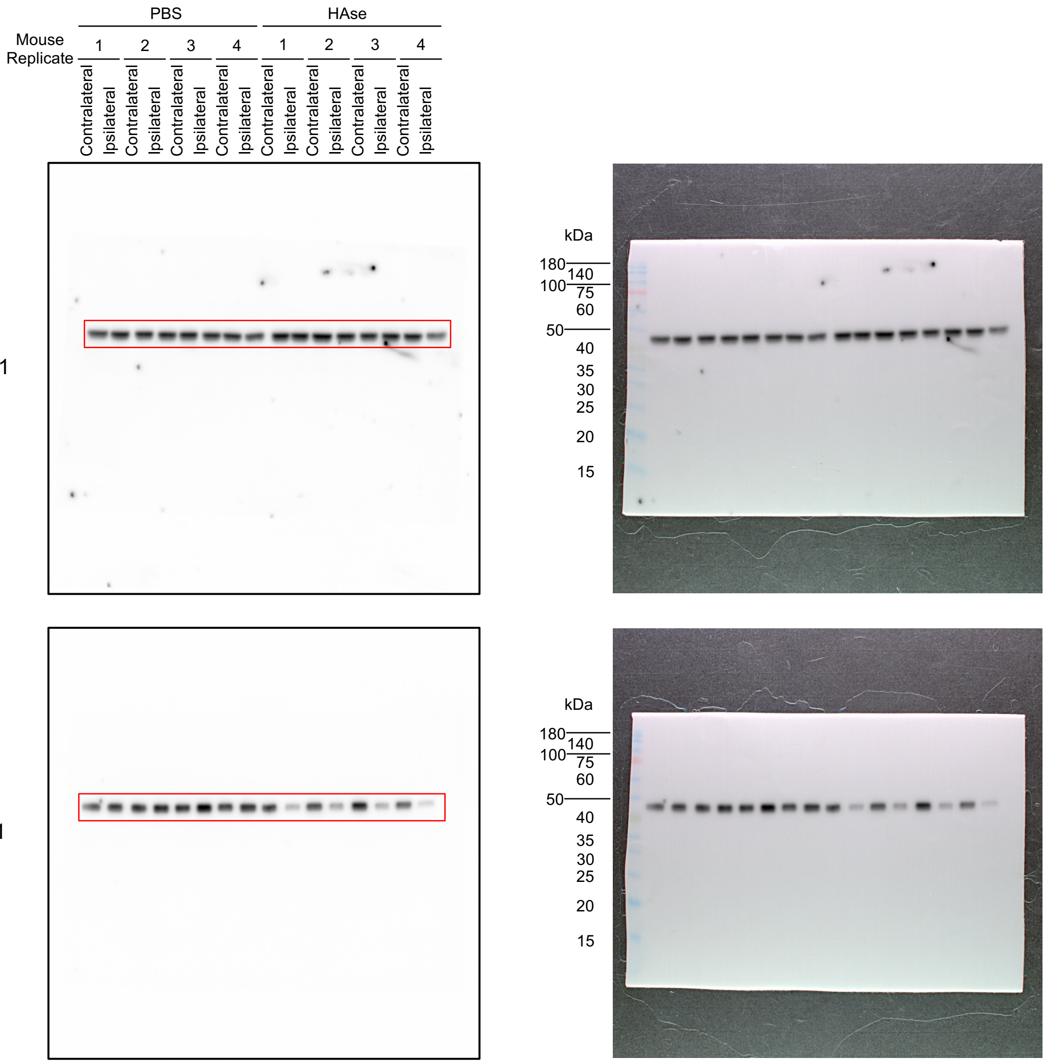

IB: HAPLN1
S2 fraction

IB: HAPLN1
P2 fraction

Figure S8A

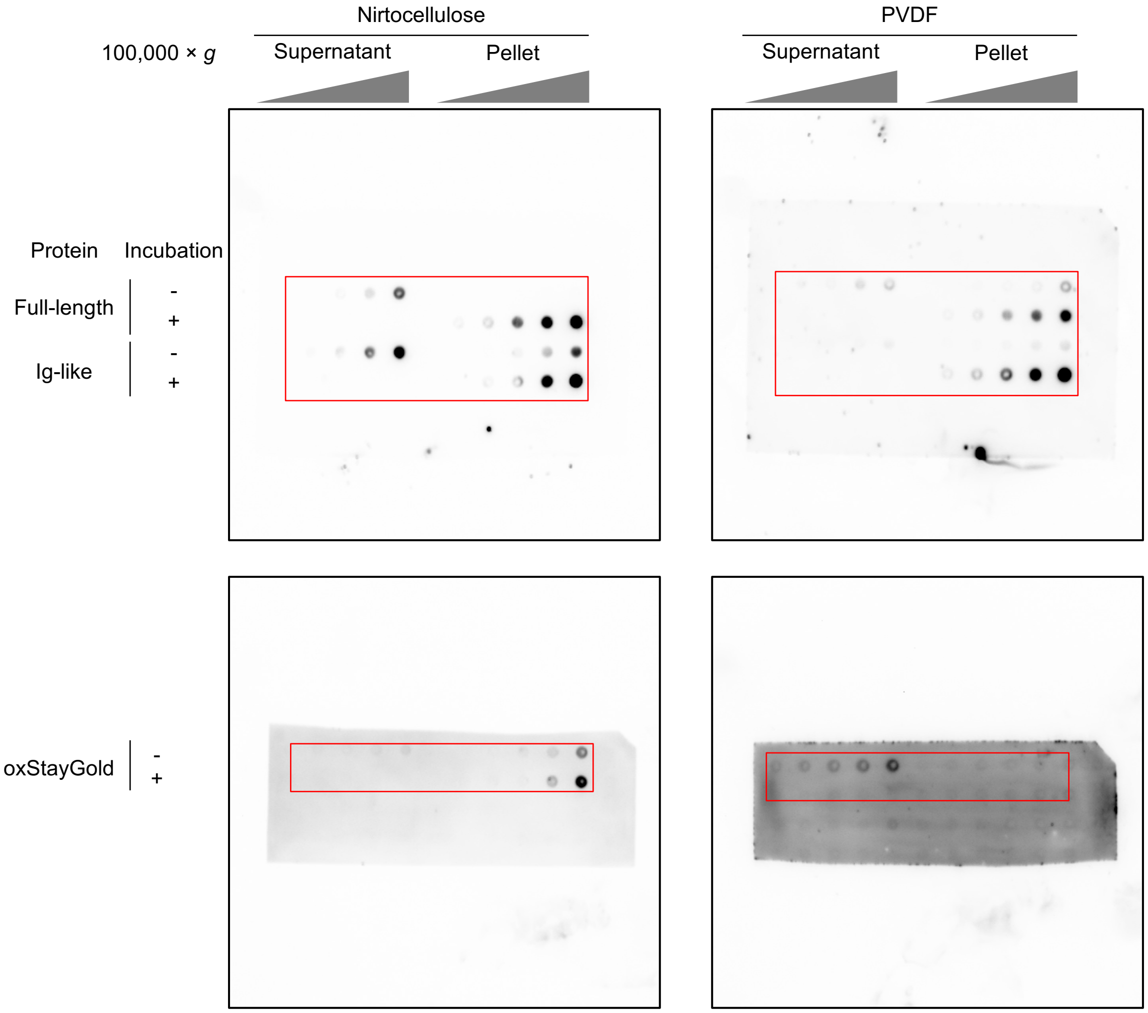
Nirtocellulose
PVDF
100,000 × g
Supernatant
Pellet
Supernatant
Pellet
Protein
Incubation
Full-length
-
+
Ig-like
-
+
oxStayGold
-
+
