# Supplementary material for: HAPLN2 forms aggregates and promotes microglial inflammation during brain aging in mice
Source: PLoS Biol. 2025 Aug 14;23(8):e3003006. doi: 10.1371/journal.pbio.3003006 (PMC12407547; doi:10.1371/journal.pbio.3003006)
Supplement: Attachment — Submitted filename: Point by point response reviewer1-3 20250619AW1 (1).docx [file pbio.3003006.s021.docx]

**Point by point responses** (*blue italics: reviewers’ comments)*

**Reviewer #1**

We sincerely appreciate Reviewer #1 for the insightful evaluation of our manuscript, as well as for this reviewer’s positive comments regarding the quality and potential significance of our work. In addition, we appreciate the reviewer’s suggestions for further strengthening our study, and we address each point below.

*“With that said, this study could be improved and made much more significant by showing that these aggregates have any physiological function. For example, do HAPLN2 knockouts have improved outcomes with increasing age?"*

Response: We thank the reviewer for highlighting the importance of investigating the physiological function of HAPLN2 aggregates *in vivo*. We agree that directly assessing their impact is crucial for understanding their biological significance. However, as previously reported, HAPLN2 knockout mice display developmental abnormalities, including impaired perinodal extracellular matrix (ECM) formation and reduced nerve conduction (Bekku et al., 2010). These phenotypes complicate the use of knockout models for evaluating the specific effects of HAPLN2 aggregates in the adult brain.

To address this limitation, we employed an alternative approach. Specifically, we administered hyaluronidase—an enzyme we demonstrated to reduce sarkosyl-insoluble HAPLN2 aggregates in the P2 fraction—into the cerebellum of nine-month-old mice and evaluated their motor function three months later, at 12 months of age (Figs 6I–P). Mice treated with hyaluronidase exhibited significantly improved motor performance compared to PBS-injected controls. Given that age-related motor deficits are often associated with cerebellar dysfunction (Radulescu et al., 2021), our findings suggest that HAPLN2 aggregates, or HAPLN2 aggregates together with other ECM aggregates, may interfere with the cerebellar regulation of motor function. We have now clarified these points in the revised manuscript (pages 18 and 19, lines 481–497). We are grateful to the reviewer for prompting us to highlight this important aspect of our study.

*“Although the authors focus on HAPLN2, perhaps there are many other proteins that are equally pathogenic. It would be interesting to know what else is in those aggregates - the authors may already know based on their mass spectrometry results.”*

Response: We appreciate the reviewer’s insightful suggestion regarding the potential pathogenicity of other age-dependent protein aggregates in addition to HAPLN2. To address this point, we further analyzed our mass spectrometry data and focused on additional proteins such as aggrecan core protein (ACAN) and HAPLN1. Immunohistochemical analysis revealed that, with aging, the area occupied by HAPLN1-positive puncta not colocalizing with ACAN increased (Figs S4A–C). Furthermore, *in vitro* hyaluronidase treatment did not solubilize HAPLN1 in the sarkosyl-insoluble P2 fraction (Figs 2C–F). These findings indicate that HAPLN1 also forms proteinaceous aggregates and may contribute to age-related pathology, similar to HAPLN2. Additionally, we provided a list of the top proteins identified in the sarkosyl-insoluble P2 fraction by mass spectrometry in S3 Table. We have included these results of their potential significance in the revised manuscript (page 13, lines 331–338).

*“Although the experiments showing that microglia are central to the process, there are no experiments that show the accumulation of HAPLN2 is biologically significant. I strongly suspect it is, but there aren't any experiments to confirm this.”*

Response: We fully understand and appreciate the reviewer’s concern regarding the lack of direct evidence for the biological significance of HAPLN2 accumulation. To address this important point, we have already taken the following two approaches:

1. *In vitro* experiments: We added recombinant HAPLN2 oligomers and aggregates to cultured microglial cells and observed activation markers characterized by increased expression of inflammatory cytokines (Figs 8B and 8C).

2. *In vivo* experiments: We injected recombinant HAPLN2 oligomers and aggregates into mouse hippocampus and cerebellum and detected microglial activation at the injection site by Iba1 staining and the expression of inflammatory cytokines (Figs 8D and 8E).

These results suggest that HAPLN2 aggregates can activate microglia both *in vitro* and *in vivo*. However, we agree that these findings remain correlative and do not establish a causal role for HAPLN2 in age-related neuroinflammation or motor decline.

To provide stronger evidence, we believe the following additional experiments are essential:

3. Genetic manipulation: Conditional overexpression or knockdown/knockout of HAPLN2 at appropriate ages to examine its effects on microglial activation and motor behavior.

4. Pharmacological intervention: To test whether microglial inhibition or depletion (e.g., via CSF1R inhibitors) attenuates HAPLN2-induced inflammation.

5. HAPLN2 removal: To assess whether degradation or removal of HAPLN2 (e.g., by hyaluronidase) suppresses microglial activation.

We fully recognize that these experiments are crucial for elucidating the biological significance of HAPLN2 accumulation and clarifying the underlying causal relationships. However, due to their technical complexity and time constraints, they could not be completed within the current revision period. We have clearly stated this limitation in the revised manuscript (pages 26–27, lines 712–720), and we are prioritizing these approaches in our ongoing work.

We have revised the manuscript to clearly state that our current findings support a potential link between age-related HAPLN2 aggregation and microglial activation, but further studies are needed to establish causality. These revisions are reflected in the revised manuscript (pages 26–27, lines 712–720).

Despite this limitation, we believe our current findings provide strong associative evidence linking age-related HAPLN2 aggregates and microglial activation. Recent studies have demonstrated that chronic microglial activation in the cerebellum contributes to motor dysfunction (Gyengesi et al., 2019; S. T. Xie et al., 2023). In this context, we consider it an important future direction to examine whether HAPLN2-induced neuroinflammation contributes to age-associated motor decline, and whether its clearance improves function.

Taken together, our current findings lay a critical foundation for future studies aimed at elucidating the pathological significance of HAPLN2 accumulation and its role in neurodegenerative processes.

*Minor comments:*

*“1. Line 240: granule cell layer, not granular layer”*

Response: We have corrected "granular layer" to "granule layer" (page 11, line 261 in the revised manuscript) as suggested.

*“2. Line 275: Caspr is a component of the paranodal junction, not node. HAPLN2 is localized to the node between the flanking paranodal junctions labeled by Caspr.”*

Response: Thank you for this precise comment regarding the localization of Caspr and HAPLN2 on Line 275. We have revised the manuscript to accurately reflect that Caspr is a component of the paranodal junction and that HAPLN2 is localized to the node of Ranvier, situated between the paranodal junctions labeled by Caspr (page 12, line 297 in the revised manuscript).

**Reviewer #2**

We are very grateful to Reviewer #2 for the careful reading of our manuscript, the positive overall evaluation, and for providing insightful comments and constructive suggestions for improvement. We particularly appreciate the reviewer's assessment of our study as "interesting and well-executed" and their support for its publication in *PLoS Biology* following minor revisions. The feedback provided has been invaluable in further refining our paper. We have addressed each of the reviewer's points in detail below, and we believe these revisions have substantially strengthened the manuscript.

*"One obvious question that is not discussed is what happens to the mice injected with hyaluronidase. Do they display any change in behavior compared to controls (e.g., better locomotion)? An extended behavioral or locomotion assessment would be beyond the scope of this study, but it would be very interesting to know (even if the results were negative)."*

Response: We thank the reviewer for raising this important question regarding behavioral changes in mice injected with hyaluronidase.

As noted in our response to Reviewer #1, mice that received cerebellar injections of hyaluronidase showed improved motor skills compared to the control group. In the open field test, this was evidenced by an increased walking distance and a decrease in meander (Figs 6I–P). These results suggest that the clearance of age-associated protein aggregates, including HAPLN2, by hyaluronidase, may help mitigate age-related motor dysfunction.

These findings support the physiological significance of HAPLN2 aggregation and provide a foundation for future studies to elucidate the causal relationship between such aggregation and age-related functional decline. We have clarified these points in the revised manuscript (pages 18 and 19, lines 481–497).

*“It was unclear why the quantification of the pellet was done using a label-free approach while the soluble fraction was analyzed by TMT. This makes the direct comparison of ratios more difficult (e.g., TMT is known for compressing differences). “*

Response: We appreciate the reviewer’s thoughtful comment regarding our approach to proteomic quantification. We agree that using different quantification methods, employing a label-free strategy for the sarkosyl-insoluble P2 fraction and TMT labeling for the PTS-soluble fraction, could complicate direct dataset comparisons, particularly considering potential TMT ratio compression effects.

To address this concern, we have reanalyzed the PTS-soluble fraction using a label-free quantification method instead of TMT, ensuring a consistent analytical approach with that used for the insoluble fraction. In this reanalysis, proteins with more than a two-fold increase in aged mice compared to young mice were considered significant. These results are now presented in the revised Figure 1C. We believe this reanalysis addresses the comparability issue of the datasets and clarifies the significance threshold. These methodological updates are now described in the revised manuscript (page 7, lines 149–158) and detailed in the Methods section (pages 29 and 30, lines 796–807).

*“Finally, it was unclear why only proteins showing an increase in both the insoluble and soluble fractions were considered. An increase in the insoluble fraction alone should suffice to draw interest, and the authors should share some information about these proteins (e.g., GO analysis). “*

Response: Regarding the reviewer's comments on our proteomic quantification strategy, we agree that our rationale for integrating results from both the sarkosyl-insoluble and PTS-soluble fractions was not sufficiently explained in the original manuscript. The sarkosyl-insoluble fraction is highly enriched in aggregated proteins, whereas the PTS-soluble fraction represents the bulk proteome (Fig S1).

Initially, we prioritized proteins that increased in both fractions, reasoning that such proteins may represent not only aggregation but also accumulation due to impaired clearance. This dual increase was also advantageous for downstream validation such as immunohistochemistry, where distinguishing aggregated from native protein can be difficult if total protein levels are unchanged. However, we agree that proteins that aggregate without a change in total abundance are of significant biological interest and may be functionally relevant to age-related pathology.

To address the reviewer’s concern directly, we performed GO analysis on proteins enriched only in the sarkosyl-insoluble fraction, defined as those with a fold change (aged/young) >5.0 and p < 0.01 (Fig 1B). This analysis revealed strong enrichment of GO terms such as “hyaluronic acid binding” and “glycosaminoglycan binding,” highlighting key ECM proteins including ACAN, HAPLN1/2, and VCAN. These proteins showed an increase in both proteomic analyses as listed in Figure 1D, confirming that our filtering strategy using soluble fraction data did not lead to the exclusion of critical aggregating candidates.

We have clarified this point in the revised manuscript (page 6, lines 136–144; page 7, lines 154–158), and we appreciate the reviewer’s suggestion, which has helped us refine both our data interpretation and the manuscript.

*“The complete list of quantified proteins should be provided, along with their identification scores, the number of peptides and signal intensities. The raw data should also be deposited in shared platform. Some additional details should be included in the method section to better explain how the quantification was performed. For instance, was the data normalized, and were missing values imputed in the label-free quantification? Did the authors notice differences in the amount recovered from each pellet fraction? “*

Response: We thank the reviewer for these constructive suggestions. We have revised the manuscript to improve the transparency of our proteomic data as follows:

Data availability: As requested, we have provided comprehensive lists of all quantified proteins (including scores, peptide counts, and intensities) in new supplementary tables (S1 and S4 Tables are related to Figure 1A and 1C, respectively). The raw mass spectrometry data have been deposited in the ProteomeXchange/jPOST repository with the accession numbers JPST003565 and PXD060043 for Figure 1A and JPST003863 and PXD064908 for Figure 1C (page 8, lines 187–189).

Quantification details: We have added details on our label-free quantification to the Methods section (page 29, lines 789–794 of the revised manuscript). Briefly, data were normalized using the "Total peptide amount" method, and missing values were imputed using the "MinDet" method using the MSnbase library in the Bioconductor R package. This method replaces missing values with random values lower than the minimum observed in each sample, assuming that missing values are likely below detection limits. We have provided tables containing the abundance values after imputation (S2 and S5 Tables are related to Figure 1A and 1C, respectively), as well as the calculated average protein quantities and associated *p*-values for both young and aged mice (S3 and S6 Tables are related to Figure 1A and 1C, respectively).

Protein recovery: We found no significant difference in the total protein recovered from the sarkosyl-insoluble fractions between young and aged mice. This data has been added as a new supplementary figure (Supplementary Figure 1 for Reviewer #2).

We thank the reviewer for their valuable feedback, which has significantly contributed to improving the manuscript.

Supplementary Figure 1 for Reviewer #2

Protein concentration of the sarkosyl-insoluble P2 fraction between young (2-month-old) and aged (24-month-old) mouse brains. Related to Figure 1A. Protein concentration was measured using the protein assay BCA kit (06385-00; Nacalai Tesque), and the absorbance was read at 560 nm using a GloMax® plate reader (Promega). n = 5. Error bars represent mean ± S.E.M. *P*-value by two-tailed Student’s *t*-test. ns = not significant.

*“It was unclear whether results obtained using nitrocellulose vs. PVDF membranes are expected to differ. If so, some brief information should be added to explain this. For instance, ACAN did show an increase in signal in tissue derived from older mice when using PVDF but not with the nitrocellulose membrane. “*

Response: We are grateful to the reviewer for highlighting this important point and regret that our previous explanation did not provide sufficient clarity.

As illustrated in the schematic (Supplementary Figure 2 for Reviewer #2), we intentionally used a stacked, dual-membrane system to separate proteins based on their physical size(Laferrière et al., 2022). Specifically, the top nitrocellulose membrane, with its larger 0.45 µm pores, physically traps large protein assemblies. Any smaller soluble proteins that pass through are subsequently captured by the bottom PVDF membrane, which has smaller 0.22 µm pores and high binding affinity.

This setup allows for a clear interpretation of our findings. For instance, the age-dependent increase of HAPLN1/2 on the top nitrocellulose membrane suggests its incorporation into large assemblies. In contrast, ACAN, as you noted, was retained only on the bottom PVDF membrane, indicating it exists in a smaller, soluble form that increases with age.

We have now clarified these points (page 9, lines 205–211 of the revised manuscript) and expanded the Methods section (page 31, lines 843–853 of the revised manuscript) to better explain how the dual membrane filter trap assay was performed. We appreciate this valuable feedback.

Supplementary Figure 2 for Reviewer #2

Schematic diagram of a dual-membrane filter trap assay using a combination of nitrocellulose and PVDF membranes.

*“Line 236: The data in Fig. 3A show good detection in the cerebellum, but the authors cannot state that this is predominant without comparison to other brain regions. “*

Response: We agree with the reviewer that using the term "predominant" was not justified without comparison to other brain regions.

To address this, we performed additional immunohistochemistry for HAPLN2 and myelin basic protein (MBP) across multiple brain regions in young and aged mice (newly added as Fig S2A). Our new data confirmed that while HAPLN2 aggregates increase with age in other myelinated areas (e.g., corpus callosum, brainstem), the accumulation is indeed most prominent in the cerebellum. This analysis revealed that with aging, HAPLN2-positive puncta increased in several highly myelinated regions, including the corpus callosum and brainstem, in addition to the cerebellar white matter. However, consistent with previous reports on HAPLN2 expression (Oohashi et al., 2002), both our immunoblotting (Fig 3A) and new immunohistochemistry data (Fig S2A) demonstrate that the cerebellum exhibited the most prominent accumulation of HAPLN2 aggregates.

Given this observation, and considering that age-related motor deficits associated with cerebellar dysfunction have been reported (Radulescu et al., 2021), we focused our analysis on HAPLN2 aggregation in the cerebellar white matter to explore its potential physiological relevance.

Based on these new findings, we have revised the text to state that the most prominent accumulation was observed in the cerebellum and have clarified our rationale for focusing on this region (pages 10 and 11, lines 257–263).

*“Could the authors perform ThT or Nile Red staining of the cerebellum to determine whether HAPLN2 forms amyloid-like aggregates? “*

Response: We appreciate the reviewer’s suggestion regarding the use of Thioflavin T (ThT) or Nile Red staining to assess whether HAPLN2 forms amyloid-like aggregates in the mouse brain. In response, we performed ThT staining on cerebellar sections from both young and aged wild-type mice. However, we did not observe clear co-localization between HAPLN2 and ThT signals. This may be due to the well-documented non-specific binding of ThT to a wide variety of β–sheet–rich proteins in the brain, such as keratins (Ly et al., 2011), which can obscure the detection of proteins like HAPLN2 that may only partially exhibit ThT or Nile Red positivity *in vitro*. Additionally, Nile Red staining often produces high background signals because of its strong affinity for lipids (Teo et al., 2021). Therefore, we conclude that the presence and structural characteristics of potential cross-β-sheet conformations of HAPLN2 *in vivo* remain unresolved and represent important questions for future investigation.

*“For Figures 6B and 8D, insets with enlargements of the images should be provided to better illustrate the reduction of HAPLN2 aggregation and the increase in microglial cells, which are not evident in the provided images.? “*

Response: We thank the reviewer for this helpful suggestion. In response, we have added enlarged views to each figure to improve clarity. Specifically, a magnified inset has been incorporated into the main panel of Figure 6B, and an enlarged image corresponding to Figure 8D has been provided as Figure S8C in the supplementary information.

**Reviewer #3**

We are sincerely grateful to Reviewer #3 for their exceptionally thorough review and scrupulous attention to detail. The valuable insights provided have been instrumental in helping us to significantly strengthen the manuscript. We have carefully addressed each point and provide our point-by-point responses below.

*“What is the rationale for using a duplicated approach to identify insoluble proteins? The protocol for processing mouse brain tissue was very similar for both proteomics and mass spectrometry approaches, yet there is a marked discrepancy between the sarkosyl-insoluble proteins identified by these methods. What could explain this inconsistency? “*

Response: The reviewer raises an important question regarding our dual fractionation approach and the rationale for our subsequent protein prioritization. Our primary goal was to identify proteins that not only form sarkosyl-insoluble aggregates with age but also show an increase in their overall abundance. While we acknowledge that proteins aggregating without a change in total abundance are also highly interesting candidates, those that show an increase in both measures offer a key advantage for downstream validation. Specifically, for techniques like immunohistochemistry, an increase in total abundance makes it easier to distinguish signals from aggregated forms versus native proteins. Furthermore, as our study aimed to investigate the decline in brain clearance mechanisms, we used these two different fractionation methods to focus on proteins whose total levels increase, making them particularly relevant targets.

Regarding the discrepancy observed between the two mass spectrometry datasets, we believe there are two main contributing factors. First, as also noted by Reviewer #2, our original analysis used TMT-based proteomics for the PTS-soluble fraction, which is known to compress fold-change ratios compared to the label-free method. To address this, we reanalyzed the PTS-soluble fraction using label-free proteomics, and these new data are presented in Figure 1C. Second, the sample compositions are inherently different. The sarkosyl-insoluble fraction is specifically enriched for protein aggregates (100,000 × *g* pellet), whereas the PTS-soluble fraction represents a much bulk proteome (15,000 × *g* supernatant). This fundamental difference also contributes to the observed discrepancies.

*“The criteria for selecting proteins for further study are not sufficiently clear. For instance, Cystm1 had an abundance aged/young mouse ratio of 58:1 but was not examined further. The authors should clarify their selection criteria. “*

Response: We appreciate the reviewer highlighting the need for clearer selection criteria. Indeed, proteins such as Cystm1, which shift from a soluble to an aggregated form with age while maintaining a constant total abundance, are biologically intriguing, and we observed several similar cases.

However, for our downstream analyses, we prioritized proteins that not only aggregate with age but also show an increase in total abundance. This was because these proteins allow for a clearer distinction between soluble and aggregated forms and facilitate the tracking of aggregation progression over time. Furthermore, as our study aimed to investigate the link between protein aggregation and the age-related decline in clearance systems (e.g., microglia and the glymphatic system), proteins with increasing abundance were considered more relevant targets.

Additionally, our GO analysis (Fig 1B) highlighted an enrichment of extracellular matrix-related proteins (e.g., ACAN, HAPLN1/2) associated with "hyaluronic acid binding," suggesting that the extracellular matrix undergoes significant age-related changes.

Taking these points into consideration, as well as the availability of reliable antibodies, we selected ACAN and HAPLN1/2 for further validation in the experiments shown in Figure 2 and beyond.

*“If ACAN did not form sarkosyl-insoluble aggregates, why was it identified in the proteomics assay? “*

Response: We thank the reviewer for raising this excellent point. Our hypothesis is that ACAN, while soluble in sarkosyl itself, is co-isolated in the insoluble fraction through its strong association with high-molecular-weight hyaluronan, a component known to be resistant to this solubilization procedure (Sugitani et al., 2021).

To directly test this hypothesis, we treated the sarkosyl-insoluble P2 fraction with hyaluronidase. This experiment confirmed that ACAN was subsequently released into the sarkosyl-soluble supernatant (S3 fraction), as shown in Figure 2D-F. This result strongly supports our model that ACAN's presence in the insoluble fraction is due to its binding with hyaluronan.

*“The presence of beta-sheet conformations should be assessed using a technique other than fluorescence. Circular dichroism, a well-established method for detecting beta-sheet structures in amyloid-beta aggregates, would be more appropriate (e.g., Harada et al. Biopolymers 2011).“*

Response: We agree with the reviewer’s suggestion that the β-sheet structures detected by ThT and Nile Red staining should be supported by an orthogonal method. In response, we analyzed the secondary structure of our recombinant HAPLN2 proteins (in both soluble and low pH-induced aggregated states) using circular dichroism (CD) spectroscopy. The results showed that while the soluble form of HAPLN2 exhibited a positive ellipticity peak at 190 nm, the aggregated form showed a shift to 200 nm. Moreover, structural prediction using BeStSel indicated an increased β-sheet content in the aggregated form. These CD spectroscopy findings are consistent with our observations in Figure 5A–E, where HAPLN2 aggregation correlates with increased ThT and Nile Red positivity. We have now clarified these points in the revised manuscript (page 16, lines 419–428). We are grateful for the reviewer's constructive comment. This orthogonal validation using CD spectroscopy provides more robust support for our conclusions and has substantially strengthened our study.

*“How can the authors be certain that aggregated HAPLN2 exists as isolated deposits/puncta rather than as a component of damaged myelin? Myelin declines substantially with aging in mice (Hill et al., Nat Neurosci 2018). Staining brain tissue for damaged myelin (e.g., with LFB or a similar method) alongside HAPLN2 could help assess colocalization. “*

Response: The reviewer’s suggestion that HAPLN2 aggregation may be associated with age-related myelin damage is very insightful. To investigate this possibility, we performed co-immunostaining of HAPLN2 with MBP (a myelin marker) and calbindin 1 (a Purkinje cell axon marker) in 3-month-old and 18-month-old mice, based on a previous study indicating that age-dependent demyelination is evident by 18 months of age (Parandavar et al., 2024). We interpret that while the loss of the MBP signal indicates demyelination, the gain of calbindin 1 signal serves as a complementary indicator because the loss of the myelin sheath likely unmasks the underlying Purkinje cell axons, increasing their accessibility to the antibody. In our hands, fluorescence immunostaining provided clearer visualization of HAPLN2 puncta than DAB staining, which is a common choice for immunohistochemistry in studies involving LFB staining, so we opted for this method for the following analysis:

1. Age-related demyelination

2. HAPLN2 aggregation in demyelinated versus less-demyelinated regions in aged mice

As shown in our new data (Figs S3A–C), aged mice exhibited increased MBP-negative voids (indicative of demyelination) with a corresponding increase in calbindin 1 staining.

However, we found no clear correlation between myelin damage and the accumulation of HAPLN2-positive puncta in the brains of aged mice. This was determined by comparing HAPLN2 deposit areas between regions with clear demyelination (decreased MBP staining and increased calbindin 1 labeling) and adjacent regions with less demyelination (Figs S3D and S3E).

Therefore, we suggest that a critical next step toward understanding the relationship between HAPLN2 accumulation and myelin damage would be to analyze their spatiotemporal correlation. This would involve determining the precise onset of both phenomena and assessing their colocalization at various stages of aging. We recognize this as a significant undertaking that represents an important direction for future work, which we now discuss in the revised manuscript (pages 12 and 13, lines 307–330).

*“It is surprising that no HAPLN2 aggregates were observed in other heavily myelinated brain regions, such as the corpus callosum. What might explain this? “*

Response: We agree with the reviewer that our initial analysis of HAPLN2 aggregation outside the cerebellum was insufficient. To address this, we have now included immunohistochemistry images of HAPLN2 and the myelin marker MBP across the whole brain (Fig S2A). These data show that, in addition to the cerebellum, HAPLN2-positive puncta also increased in number and size with age in other heavily myelinated regions, such as the brainstem and corpus callosum. Therefore, we believe that HAPLN2 aggregation is indeed associated with highly myelinated regions in the aging brain.

However, consistent with previous reports (Oohashi et al., 2002), both our immunoblotting (Fig 3A) and new immunohistochemistry data (Fig S2A) demonstrate that the cerebellum exhibited the most prominent accumulation of HAPLN2 aggregates. Given this observation, and considering reported age-related motor deficits associated with cerebellar dysfunction (Radulescu et al., 2021), we focused our analysis on the cerebellar white matter to explore its potential physiological relevance. We have now clarified these points in the revised manuscript (pages 10 and 11, lines 257–263).

*“Could HAPLN2 aggregates colocalize with late endosomes and lysosomes? The punctate distribution might suggest colocalization with these organelles. The authors could assess this by staining for LAMP1 alongside HAPLN2. “*

Response: This is an insightful comment. Regarding the possibility of lysosomal localization, our data in Figure 3E show that HAPLN2 colocalizes with HABP, a marker of the extracellular matrix, suggesting that HAPLN2 is predominantly localized extracellularly rather than within lysosomal compartments.

Furthermore, under our experimental conditions using aged mice (18–24 months old), we did not observe the presence of LAMP1-positive disease-associated microglia (DAMs), which are typically seen in models of neurodegenerative disease (Safaiyan et al., 2021). We also found that HAPLN2 aggregation begins to appear in middle-aged mice (12 months old), a time point at which DAMs are generally absent. These observations suggest that the clearance of HAPLN2 aggregates is not necessarily dependent on LAMP1-positive DAMs.

Nevertheless, elucidating the precise mechanisms by which microglia clear HAPLN2 aggregates—whether through phagocytosis, peptidase activity, or digestive exophagy—remains an important direction for future investigation. To address this, it will be necessary to define the time window during which microglial activation occurs in the vicinity of HAPLN2 aggregates.

*“The role of hyaluronic acid in HAPLN2 aggregation remains unclear in the present study. Hyaluronic acid promoted HAPLN2 aggregate formation (Fig 4) but did not appear necessary for aggregate maintenance (Fig 2). However, hyaluronidase treatment in mice promoted HAPLN2 aggregate clearance in the cerebellum (Fig 6). How do the authors reconcile these observations? “*

Response: We appreciate the reviewer’s insightful comment. We acknowledge that our explanation regarding the role of hyaluronic acid was not sufficiently clear. We have made the following clarifications:

First, as shown in Figure 5, hyaluronic acid promotes the aggregation of recombinant HAPLN2. HAPLN2 has a theoretical pI of 9.04 and carries a strong net positive charge (+11.2) at physiological pH. We hypothesize that the addition of the polyanionic compound hyaluronic acid neutralizes this charge, thereby promoting aggregate formation (Lin et al., 2020).

Regarding the hyaluronidase experiments, the different outcomes in Figures 2 and 6 can be explained by the different experimental conditions. In Figure 2, hyaluronidase was applied to sarkosyl-insoluble P2 fractions from brain homogenates, where physiological clearance mechanisms are inactive. In contrast, in Figure 6, hyaluronidase was injected into live mice where clearance mechanisms like the glymphatic system were functioning.

Our interpretation is as follows: In Figure 2, hyaluronidase treatment did not solubilize the HAPLN2 in the P2 fraction. This suggests that while hyaluronidase can break down functional ECM components, the HAPLN2 had formed structurally rigid, irreversible aggregates resistant to this enzymatic action. In Figure 6, however, hyaluronidase injection into the cerebellum likely expanded the extracellular space and promoted glymphatic clearance (Godin et al., 2016; L. Xie et al., 2013). Thus, while the aggregates may not have been directly solubilized, their clearance from the brain was enhanced.

Taken together, our findings suggest a dual role for hyaluronic acid: it promotes HAPLN2 aggregation and also contributes to the inhibition of aggregate clearance. Therefore, age-related increases in hyaluronic acid may both facilitate the formation of rigid HAPLN2 aggregates and impair their removal.

Supplementary Figure 3 for Reviewer #3

Schematic model for the dual role of hyaluronic acid in the aggregation and clearance of HAPLN2.

HAPLN2 carries a net positive charge at physiological pH (up). Its interaction with the polyanionic hyaluronic acid is thought to neutralize this charge, thereby promoting the formation of large, structurally rigid HAPLN2 aggregates (middle). The dense extracellular matrix formed by these aggregates likely inhibits their clearance from the brain (bottom). *In vivo*, the enzymatic degradation of hyaluronic acid by hyaluronidase injection can expand the extracellular space, which enhances the removal of HAPLN2 aggregates, likely via the glymphatic system. This model suggests that hyaluronic acid plays a dual role in both promoting HAPLN2 aggregation and impairing its subsequent clearance.

*“The authors suggest that reducing hyaluronic acid increases extracellular space, facilitating microglial access to HAPLN2 aggregates. However, this claim lacks direct evidence. The observed effect could be due solely to the reduction of hyaluronic acid, which influences aggregate formation (Fig. 2 and point 8 above) “*

Response: The reviewer correctly points out that our interpretation of hyaluronidase facilitating microglial access may have been an overstatement. To investigate the clearance mechanism further, we compared HAPLN2 protein levels in the cerebrospinal fluid (CSF) of mice with and without intracerebellar hyaluronidase injection (Fig 6G). We found that the amount of HAPLN2 in the CSF increased following hyaluronidase administration.

This result suggests that the reduction of HAPLN2 following hyaluronidase injection is more likely due to the enhancement of glymphatic clearance rather than improved microglial access. However, as the reviewer also noted, the glymphatic system primarily clears small, soluble waste. It is possible that the larger HAPLN2 aggregates were solubilized through an unknown mechanism before being cleared into the CSF, but clarifying this point remains a task for future studies. We have now clarified these points in the revised manuscript (page 18, lines 469–480).

*“Increased HAPLN2 aggregation upon microglia depletion might result from impaired myelin clearance in the absence of these cells, rather than HAPLN2 clearance per se. (see point 5). The authors might want to address that. “*

Response: The reviewer’s suggestion that HAPLN2 accumulation upon PLX3397 treatment might be secondary to impaired myelin clearance is indeed insightful. Previous work has reported that while PLX3397 can suppress myelin damage in the cuprizone-induced multiple sclerosis model, PLX3397 treatment alone does not affect myelin integrity in healthy mice (Zirngibl et al., 2022). To confirm this in our model, we performed immunostaining for MBP and calbindin on the same cohort of mice shown in Figure 7 (treated with PLX3397 from one to three months of age) (Fig S7). We did not observe any significant change in the calbindin-positive area upon PLX3397 treatment, suggesting that significant myelin damage did not occur at this young age. Furthermore, considering our findings in 18-month-old mice that there was no correlation between demyelination and HAPLN2 accumulation (Fig S3), these findings together support the idea that HAPLN2 accumulation following PLX3397 treatment is independent of microglia-mediated clearance of damaged myelin. Nevertheless, the precise mechanism by which microglia are involved in HAPLN2 degradation remains to be elucidated. We have now clarified these points in the revised manuscript (page 20, lines 532–542).

*“When quantifying HAPLN2 in mouse brain sections, the authors analyze individual puncta or aggregates (Figs. 3, 4, 6, 7, 9), leading to a large N. This constitutes pseudoreplication. The smallest independent unit should be at least an individual brain area field. 6-8 fields within cerebellum or hippocampus should be imaged per mouse brain section, quantified and used as N for statistical analyses. “*

Response: We appreciate the reviewer's careful observation. The reviewer makes a valid point regarding the potential for pseudoreplication when analyzing individual puncta. In response, we have revised our analysis for all graphs comparing puncta size in our immunohistochemistry data. We now use the mean puncta area per field of view as the unit for analysis, with each field representing a technical replicate and each mouse representing a biological replicate.

*“In Figure 9, the 60-69-year-old donor exhibits almost no HAPLN2 aggregates, whereas the 80-89-year-old donor shows extensive aggregation. However, the quantification in panel B does not align with the presented images. The authors should address this discrepancy. “*

Response: We agree with the reviewer that there appears to be a discrepancy between the images in Figure 9A and the puncta size quantification in the original Figure 9B. To address this, we have now also quantified the number of HAPLN2-positive puncta (new Figure 9B). This new analysis reveals a dramatic increase in the number of puncta in the 80–89-year-old donor compared to the 60–69-year-old donor. Therefore, we believe that the age-related changes in HAPLN2 accumulation shown in Figure 9A reflect changes not only in puncta size but also, and more dramatically, in their number. We have now clarified these points in the revised manuscript (page 23, lines 621–624)

*“The Discussion section requires further clarification, specifically: a. Lines 522-524: What is the relevance of amyloid-beta and tau in this context? “*

Response: The reviewer correctly notes that the relevance of sarkosyl-insoluble fractions to amyloid-beta (Aβ) and tau was not sufficiently explained. Sarkosyl-insoluble fractionation is a widely used method to enrich for known pathological protein aggregates, as studies have shown that Aβ and tau specifically accumulate in the sarkosyl-insoluble fractions of Alzheimer's disease brains (Hales et al., 2016). We applied this established methodology with the hypothesis that, even in non-disease physiological aging, other proteins might similarly form insoluble aggregates. We have now clarified this rationale in the Discussion (page 24, lines 641–643).

*“Why are HAPLN2 aggregates absent in the corpus callosum of aged mice? “*

Response: As we described in our response to Comment 6, our further analysis confirmed that HAPLN2 in fact forms aggregates in other brain regions during aging, including the brainstem and corpus callosum. We have now added this point to the Discussion to correct our initial oversight (page 24, lines 646–650). We thank the reviewer for this insightful comment, which has allowed us to improve the scientific accuracy of our manuscript.

*“Lines 546-547: Why is it significant that HAPLN2 forms aggregates via protein-protein interactions rather than complexing with hyaluronic acid? “*

Response: The reviewer's question is reasonable, and we acknowledge our explanation was insufficient. Although physiological interactions between hyaluronic acid and Link domains in HAPLN2 are known, we focused on the protein-protein interactions between HAPLN2 molecules because the β-sheet-rich structures we observed (Figure 5) appeared to be specific to the aggregated form of the protein, suggesting these interactions are key drivers of pathological aggregation. However, we recognize that abnormal interactions between HAPLN2 and hyaluronic acid might also contribute to aggregation, particularly under conditions where hyaluronic acid facilitates aggregate formation, reminiscent of how polyanions promote tau aggregation (Montgomery et al., 2023). We now state that this remains an important question for future investigation (page 25, lines 663–672).

*“Lines 554-557: The claim that microglia enhance HAPLN2 clearance due to increased extracellular space is unsubstantiated... large aggregates are unlikely to be cleared through the glymphatic system... “*

Response: As also addressed in our response to Comment 9, we agree that the interpretation of enhanced microglial access was an overstatement. Our additional data show that hyaluronidase injection leads to the appearance of HAPLN2 in the CSF (Fig 6G), suggesting that enhanced glymphatic clearance contributes to the observed reduction in HAPLN2. However, as the reviewer rightly points out, the glymphatic system primarily clears small, soluble waste. The mechanism for clearing the much larger HAPLN2 aggregates we observe (Fig 3) remains unclear. We have now acknowledged this as a limitation and an important subject for future investigation in the Discussion (page 25, lines 673–685).

*“Lines 563-565: A 0.1 pH unit decrease in brain acidity is unlikely to significantly contribute to HAPLN2 aggregation. Myelin damage may be a more plausible source. “*

Response: As the reviewer rightly pointed out, our suggestion that a 0.1 unit change in pH significantly contributes to HAPLN2 aggregation was likely an overstatement. We have therefore revised the manuscript to tone down the language regarding the potential contribution of brain acidification to HAPLN2 aggregation (pages 25 and 26, lines 686–694).

In accordance with the reviewer's suggestion, we have revised the manuscript as follows:

“Additionally, recombinant HAPLN2 protein efficiently formed aggregates under low pH conditions (Fig 5). Although the decrease in brain pH with age is relatively small, ranging from 6.30 to 6.00 in humans and from 6.85 to 6.70 in mice (Decker et al., 2021), it cannot be completely ruled out as a contributing factor to HAPLN2 aggregation. While this modest change in acidity alone may not be sufficient to induce aggregation, it is possible that it interacts with other aging-related factors to promote HAPLN2 aggregation.”

*“Lines 572-572: Increased CD22 expression is not the sole contributor to declining microglial phagocytic capacity. The authors should explore this in greater detail. “*

Response: We agree with the reviewer that increased CD22 expression is not the sole contributor to the age-related decline in microglial phagocytic capacity. We have now added references indicating that reduced expression of TREM2 and ABCA7 is also implicated in this decline (Hickman & El Khoury, 2014; Lyssenko & Praticò, 2021) to provide a more balanced perspective (page 26, lines 697–701).

*Minor comments:*

*“1. Paragraph beginning at line 187: Requires grammatical refinement. “*

Response: As noted in our response to Reviewer #2, Comment 4, our explanation of the roles of nitrocellulose and PVDF membranes in the filter trap assay was insufficient. In our revised text, we clarify that we employed a double-membrane approach (Laferrière et al., 2022). The upper layer was a nitrocellulose membrane (relatively low hydrophobicity, larger pore size), and the lower layer was a PVDF membrane (more hydrophobic, smaller pore size). This setup was designed so that large protein assemblies would be retained by the nitrocellulose membrane, while other proteins would pass through and bind to the PVDF membrane (pages 9 and 10, lines 205–237).

*“Endotoxin-free protein production is achieved using ClearColi or similar technology. The original paper should be cited. “*

Response: The reviewer is correct. We used the endotoxin-free *E. coli* strain ClearColi for protein production, and we have now added the appropriate citation to the original reference (Mamat et al., 2013) in the manuscript (page 21, lines 565–567).

*“Figure legends should specify the statistical analyses used. For example, the Figure 9 legend should state: "Differences in HAPLN2 aggregate load were compared using a two-tailed Student's t-test," followed by the range of p-values and statistical significance markers. “*

Response: We have revised all figure legends to clearly specify the statistical methods used. Furthermore, we have revised all relevant graphs to include appropriate statistical significance markers.

*“Line 840 (Statistics and Reproducibility): When n represents mice, SEM should be reported instead of SD. A range of p-values and statistical significance markers should be included. “*

Response: In all cases where n represents the number of mice, we have changed the error bars to represent mean ± S.E.M. We have also ensured statistical significance markers are included in all relevant graphs.

*“Line 856 (Acknowledgment section): The authors may want to include their funding sources. “*

Response: The funding sources have been provided in the submission form, as there is a designated section for this information.

*“Lines 90-92 (Introduction): PLOS Biology serves a broad audience, so biological concepts should be thoroughly explained. For example, a general reader may not be familiar with 'daf-2' and its biological significance. “*

Response: The reviewer makes an excellent point about the broad audience of *PLoS Biology*. Our explanation of the factors regulating lifespan in *C. elegans* was insufficient. We have now clarified that *daf-2* is the *C. elegans* homolog of the mammalian insulin/IGF-1 receptor and acts as a negative regulator of *daf-16*. We further explain that *daf-16* is a transcription factor for antioxidant enzymes and chaperones, and *hsf-1* is a master regulator of the heat shock response. These details have been added to the manuscript (page 5, lines 90–95).

*“Lines 74-76 (Introduction): Digestive exophagy is another extracellular mechanism by which microglia degrade aggregates and should be mentioned. “*

Response: As the reviewer rightly pointed out, digestive exophagy is another important extracellular mechanism for microglial clearance of protein aggregates. We have now included this information and the relevant citation (Jacquet et al., 2024) in the manuscript (page 4, line 75).

**References**

Bekku, Y., Vargová, L., Goto, Y., Vorísek, I., Dmytrenko, L., Narasaki, M., Ohtsuka, A., Fässler, R., Ninomiya, Y., Syková, E., & Oohashi, T. (2010). Bral1: Its Role in Diffusion Barrier Formation and Conduction Velocity in the CNS. *The Journal of Neuroscience*, *30*(8), 3113–3123. https://doi.org/10.1523/JNEUROSCI.5598-09.2010

Decker, Y., Németh, E., Schomburg, R., Chemla, A., Fülöp, L., Menger, M. D., Liu, Y., & Fassbender, K. (2021). Decreased pH in the aging brain and Alzheimer’s disease. *Neurobiology of Aging*, *101*(1), 40–49. https://doi.org/10.1016/j.neurobiolaging.2020.12.007

Godin, A. G., Varela, J. A., Gao, Z., Danné, N., Dupuis, J. P., Lounis, B., Groc, L., & Cognet, L. (2016). Single-nanotube tracking reveals the nanoscale organization of the extracellular space in the live brain. *Nature Nanotechnology 2016 12:3*, *12*(3), 238–243. https://doi.org/10.1038/nnano.2016.248

Gyengesi, E., Rangel, A., Ullah, F., Liang, H., Niedermayer, G., Asgarov, R., Venigalla, M., Gunawardena, D., Karl, T., & Münch, G. (2019). Chronic microglial activation in the GFAP-IL6 mouse contributes to age-dependent cerebellar volume loss and impairment in motor function. *Frontiers in Neuroscience*, *13*. https://doi.org/10.3389/fnins.2019.00303

Hales, C. M., Dammer, E. B., Deng, Q., Duong, D. M., Gearing, M., Troncoso, J. C., Thambisetty, M., Lah, J. J., Shulman, J. M., Levey, A. I., & Seyfried, N. T. (2016). Changes in the detergent-insoluble brain proteome linked to amyloid and tau in Alzheimer’s Disease progression. *PROTEOMICS*, *16*(23), 3042–3053. https://doi.org/10.1002/pmic.201600057

Hickman, S. E., & El Khoury, J. (2014). TREM2 and the neuroimmunology of Alzheimer’s disease. *Biochemical Pharmacology*, *88*, 495–498. https://doi.org/10.1016/j.bcp.2013.11.021

Jacquet, R. G., González Ibáñez, F., Picard, K., Funes, L., Khakpour, M., Gouras, G. K., Tremblay, M.-È., Maxfield, F. R., & Solé-Domènech, S. (2024). Microglia degrade Alzheimer’s amyloid-beta deposits extracellularly via digestive exophagy. *Cell Reports*, *43*, 115052. https://doi.org/10.1016/j.celrep.2024.115052

Laferrière, F., Claverol, S., Bezard, E., De Giorgi, F., & Ichas, F. (2022). Similar neuronal imprint and no cross-seeded fibrils in α-synuclein aggregates from MSA and Parkinson’s disease. *Npj Parkinson’s Disease*, *8*(1), 1–12. https://doi.org/10.1038/s41531-021-00264-w

Lin, Y., Fichou, Y., Zeng, Z., Hu, N. Y., & Han, S. (2020). Electrostatically Driven Complex Coacervation and Amyloid Aggregation of Tau Are Independent Processes with Overlapping Conditions. *ACS Chemical Neuroscience*, *11*(4), 615–627. https://doi.org/10.1021/acschemneuro.9b00627

Lyssenko, N. N., & Praticò, D. (2021). ABCA7 and the altered lipidostasis hypothesis of Alzheimer’s disease. *Alzheimer’s and Dementia*, *17*, 164–174. https://doi.org/10.1002/alz.12220

Mamat, U., Woodard, R. W., Wilke, K., Souvignier, C., Mead, D., Steinmetz, E., Terry, K., Kovacich, C., Zegers, A., & Knox, C. (2013). Endotoxin-free protein production—ClearColi^TM^ technology. *Nature Methods*, *10*(9), 916–916. https://doi.org/10.1038/nmeth.f.367

Montgomery, K. M., Carroll, E. C., Thwin, A. C., Quddus, A. Y., Hodges, P., Southworth, D. R., & Gestwicki, J. E. (2023). Chemical Features of Polyanions Modulate Tau Aggregation and Conformational States. *Journal of the American Chemical Society*, *145*, 3926–3936. https://doi.org/10.1021/jacs.2c08004

Oohashi, T., Hirakawa, S., Bekku, Y., Rauch, U., Zimmermann, D. R., Su, W. D., Ohtsuka, A., Murakami, T., & Ninomiya, Y. (2002). Bral1, a brain-specific link protein, colocalizing with the versican V2 isoform at the nodes of Ranvier in developing and adult mouse central nervous systems. *Molecular and Cellular Neuroscience*, *19*, 43–57. https://doi.org/10.1006/mcne.2001.1061

Parandavar, E., Shafizadeh, M., Ahmadian, S., & Javan, M. (2024). Long-term demyelination and aging-associated changes in mice corpus callosum; evidence for the role of accelerated aging in remyelination failure in a mouse model of multiple sclerosis. *Aging Cell*. https://doi.org/10.1111/acel.14211

Radulescu, C. I., Cerar, V., Haslehurst, P., Kopanitsa, M., & Barnes, S. J. (2021). The aging mouse brain: cognition, connectivity and calcium. *Cell Calcium*, *94*, 102358. https://doi.org/10.1016/J.CECA.2021.102358

Safaiyan, S., Besson-Girard, S., Kaya, T., Cantuti-Castelvetri, L., Liu, L., Ji, H., Schifferer, M., Gouna, G., Usifo, F., Kannaiyan, N., Fitzner, D., Xiang, X., Rossner, M. J., Brendel, M., Gokce, O., & Simons, M. (2021). White matter aging drives microglial diversity. *Neuron*, *109*, 1100-1117.e10. https://doi.org/10.1016/j.neuron.2021.01.027

Sugitani, K., Egorova, D., Mizumoto, S., Nishio, S., Yamada, S., Kitagawa, H., Oshima, K., Nadano, D., Matsuda, T., & Miyata, S. (2021). Hyaluronan degradation and release of a hyaluronan-aggrecan complex from perineuronal nets in the aged mouse brain. *Biochimica et Biophysica Acta - General Subjects*, *1865*(2), 129804. https://doi.org/10.1016/j.bbagen.2020.129804

Teo, W., Caprariello, A. V., Morgan, M. L., Luchicchi, A., Schenk, G. J., Joseph, J. T., Geurts, J. J. G., & Stys, P. K. (2021). Nile Red fluorescence spectroscopy reports early physicochemical changes in myelin with high sensitivity. *Proceedings of the National Academy of Sciences of the United States of America*, *118*. https://doi.org/10.1073/pnas.2016897118

Xie, L., Kang, H., Xu, Q., Chen, M. J., Liao, Y., Thiyagarajan, M., O’Donnell, J., Christensen, D. J., Nicholson, C., Iliff, J. J., Takano, T., Deane, R., & Nedergaard, M. (2013). Sleep Drives Metabolite Clearance from the Adult Brain. *Science*, *342*(6156), 373–377. https://doi.org/10.1126/science.1241224

Xie, S. T., Fan, W. C., Zhao, X. Sen, Ma, X. Y., Li, Z. L., Zhao, Y. R., Yang, F., Shi, Y., Rong, H., Cui, Z. S., Chen, J. Y., Li, H. Z., Yan, C., Zhang, Q., Wang, J. J., Zhang, X. Y., Gu, X. P., Ma, Z. L., & Zhu, J. N. (2023). Proinflammatory activation of microglia in the cerebellum hyperexcites Purkinje cells to trigger ataxia. *Pharmacological Research*, *191*. https://doi.org/10.1016/j.phrs.2023.106773

Zirngibl, M., Assinck, P., Sizov, A., Caprariello, A. V., & Plemel, J. R. (2022). Oligodendrocyte death and myelin loss in the cuprizone model: an updated overview of the intrinsic and extrinsic causes of cuprizone demyelination. In *Molecular Neurodegeneration* (Vol. 17). BioMed Central Ltd. https://doi.org/10.1186/s13024-022-00538-8
