# Supplementary material for: HAPLN2 forms aggregates and promotes microglial inflammation during brain aging in mice
Source: PLoS Biol. 2025 Aug 14;23(8):e3003006. doi: 10.1371/journal.pbio.3003006 (PMC12407547; doi:10.1371/journal.pbio.3003006)
Supplement: Attachment — Submitted filename: 250723 Point by point responses AW.docx [file pbio.3003006.s022.docx]

**Point-by-point responses** (*blue italics: reviewers’ comments)*

We sincerely appreciate the editor’s comments aimed at improving the title, ethics statement, and data accessibility of our manuscript. We are grateful for these thoughtful suggestions and address each point in detail below.

*1) TITLE: We encourage you to change to the title of your paper, to reflect that HAPLN2 not only accumulates in the aging brain, but that this has functional consequences. If you agree, we suggest you change the title to something like:

"HAPLN2 forms aggregates and promotes microglial inflammation during brain aging in mice"*

We are very grateful for the good title suggestion. The new title of the manuscript is: "HAPLN2 forms aggregates and promotes microglial inflammation during brain aging in mice" (page 1, lines 3 and 4).

*2) ETHICS STATEMENTS: Thank you for including an ethics statement related to the human and mouse studies performed here. We ask that you update the "Post-mortem brain samples" section of your methods to indicate whether the study was conducted according to the principles expressed in the Declaration of Helsinki. Please update the "Animals" section to include the specific national or international regulations/guidelines to which your animal care and use protocol adhered. Please note that institutional or accreditation organization guidelines (such as AAALAC) do not meet this requirement.*

Thank you very much for your detailed guidance on the ethics statements. In response to the minor revision comments:

1. For the post-mortem brain experiment, we have added the following statement to the Methods section (page 27, lines 740–742):

“In accordance with the Declaration of Helsinki, written informed consent was obtained from all participants prior to their passing, or from their families.”

2. For the mice experiment, we have added the following statement to the Methods section (page 28, lines 748–754):

“These experiments were conducted in strict accordance with the following Japanese laws and guidelines: the Act on Welfare and Management of Animals (Act No. 105 of October 1, 1973), the Guidelines for Proper Conduct of Animal Experiments (published by the Science Council of Japan on June 1, 2006), and the Fundamental Guidelines for Proper Conduct of Animal Experiment and Related Activities in Academic Research Institutions (published by the Ministry of Education, Culture, Sports, Science and Technology of Japan, Notice No. 71 of June 1, 2006).

*3) DATA: Thank you for providing your mass spec data on ProteomeXchange. I could not access this data. Can you provide me with a reviewer token so that i can check that it meets our reporting requirements? (sorry if I missed this somewhere!). Please note that we will require that this data be made public at the time your paper is published.*

Thank you for your comment regarding data accessibility.

We apologize for the inconvenience—the mass spectrometry data were initially inaccessible because the original announcement date had been set to a later time. We have now updated the settings so that the data is accessible to reviewers.

The underlying mass spectrometry data are available from the ProteomeXchange Consortium via the jPOST repository (https://repository.jpostdb.org/) under the following dataset identifiers:

**・**For panel (A):
Dataset IDs: JPST003565 and PXD060043
Preview link: <https://repository.jpostdb.org/preview/7594067046879a9fb8afaa>
Access key: **9730**

**・**For panel (C):
Dataset IDs: JPST003863 and PXD064908
Preview link: <https://repository.jpostdb.org/preview/13041358516879a9ed2ec8b>
Access key: **6061**

Please note that the data will be made publicly available at the time of publication, in accordance with the journal’s policy.

*4) DATA: Thank you also for providing an excel file with the underlying data for your study (S1_data). Can you please add a sentence to each figure legend, pointing readers to this file?*

Thank you very much for helping us improve the data transparency.

We have now added the following sentence to the legends of each relevant figure and supplementary figure, as appropriate: “The underlying data can be found in S1_Data.”

Specifically, this has been included in:

- Figure 1 (page 7, line 183),
- Figure 2 (page 10, line 242),
- Figure 3 (page 12, lines 367 and 368),
- Figure 4 (page 15, lines 389 and 390),
- Figure 5 (page 17, lines 452 and 453),
- Figure 6 (page 20, lines 521 and 522),
- Figure 7 (page 21, lines 560 and 561),
- Figure 8 (page 23, line 621),
- Figure 9 (page 24, lines 638 and 639),
- S3_Fig (page 48, line 1372),
- S4_Fig (page 48, line 1380),
- S5_Fig (pages 48 and 49, lines 1389 and 1399),
- S6_Fig (page 49, line 1401),
- S7_Fig (page 49, line 1411).

We hope this addresses the comment adequately.

*5) CODE: Per journal policy, if you have generated any custom code during the course of this investigation, please make it available without restrictions. Please ensure that the code is sufficiently well documented and reusable, and that your Data Statement in the Editorial Manager submission system accurately describes where your code can be found.*

Thank you for your comment regarding code availability. We would like to clarify that no custom code was generated during the course of this study.

*Journal Requirements:*

*- We do not publish any copyright or trademark symbols that usually accompany proprietary names, eg ©, ®, ™ (e.g. next to drug or reagent names). Please remove all instances of trademark/copyright symbols throughout the text, including:*

Thank you very much for your helpful comment. I have removed all ® and ™ symbols from the manuscript in accordance with the journal’s requirements.
